# Challenges in reporting pathogenic/potentially pathogenic variants in 94 cancer predisposing genes - in pediatric patients screened with NGS panels

**DOI:** 10.1038/s41598-019-57080-9

**Published:** 2020-01-14

**Authors:** Adela Chirita-Emandi, Nicoleta Andreescu, Cristian G. Zimbru, Paul Tutac, Smaranda Arghirescu, Margit Serban, Maria Puiu

**Affiliations:** 1 Center of Genomic Medicine, Medical Genetics Discipline, University of Medicine and Pharmacy “Victor Babes”, Timisoara, 300041 Romania; 2 Regional Center of Medical Genetics Timis, Clinical Emergency Hospital for Children “Louis Turcanu”, Timisoara, 300011 Romania; 3 Department of Automation and Applied Informatics, Politehnica University Timisoara, 300006 Timisoara, Romania; 4 Pediatric Department, University of Medicine and Pharmacy “Victor Babes”, Timisoara, 300041 Romania; 5 Hematology-Oncology department, Clinical Emergency Hospital for Children “Louis Turcanu”, Timisoara, 300011 Romania

**Keywords:** Cancer genomics, Risk factors

## Abstract

The benefit of reporting unsolicited findings in Next Generation Sequencing (NGS) related to cancer genes in children may have implications for family members, nevertheless, could also cause distress. We aimed to retrospectively investigate germline variants in 94 genes implicated in oncogenesis, in patients referred to NGS testing for various rare genetic diseases and reevaluate the utility of reporting different classes of pathogenicity. We used in silico prediction software to classify variants and conducted manual review to examine unsolicited findings frequencies in 145 children with rare diseases, that underwent sequencing - using a 4813 gene panel. The anonymized reanalysis revealed 18250 variants, of which 126 were considered after filtering. Six pathogenic variants (in *BRCA1,BMPR1A,FANCA,FANCC,NBN* genes) with cancer related phenotype and three unsolicited variants (in *BRCA2,PALB2,RAD50* genes) were reported to patients. Additionally, three unsolicited variants in *ATR, BLM* (in two individuals), and *FANCB* genes presented potential cancer susceptibility, were not reported to patients. In retrospect, 4.8% (7/145) of individuals in our cohort had unsolicited NGS findings related to cancer. More efforts are needed to create an updatable consensus in reporting variants in cancer predisposing genes, especially for children. Consent process is crucial to inform of both value and risk of additional genetic information.

## Introduction

Next-Generation Sequencing (NGS) for large panels of genes or exomes are increasingly and successfully used in medical management for rare diseases and cancer. Due to their design they can identify “incidental” or “unsolicited” findings that represent additional information unrelated to the indication for the test. We will use the term “unsolicited” findings, to refer to variants in disease-causing genes that are unrelated to the original rationale for testing but discovered unintentionally. The term secondary findings will be used to refer to variants in disease-causing genes that are unrelated to the original rationale for testing but are actively sought during the analysis, as recommended by the American College of Medical Genetics and Genomics (ACMG) in 2017^1^. In 2013, the ACMG has suggested a policy for the reporting unsolicited findings to patients, families and physicians, recommending that laboratories report medically actionable variants, irrespective of the indication for testing and of the patient’s age and preference^2^. These 2013 ACMG recommendations were intensely debated and even accused of “being paternalistic”^3^, and were updated and revised in 2015^4^. In Europe, a similar consensus was attempted in 2015 and stated that if a variant has minor consequences or a clinical intervention is possible, it should be reported, with genetic counselling and informed consent being essential^5^. Additionally, the European guideline for diagnostic NGS in 2016 stated that laboratories should have a clearly defined protocol for addressing unsolicited and secondary findings^6^.

Interpretation of the ACMG recommendation vary between different laboratories in United States^7,8^, while in Europe there is similar lack of consistency^5^. A recent study showed that some laboratories limit their reporting to findings that are relevant to the clinical question, while others report unsolicited findings to varying degrees^9^.

The benefit of reporting secondary findings for children are even more sensible, as they may have implications for the parents and other family members, nevertheless, they could cause distress^10^. Therefore, strategies are needed to ensure that the consent process provides appropriate information on both value and risk of additional genetic information.

In the recommended minimum list of genes selected by the ACMG committee^2^, on the basis of their medical actionability, 23 out of 56 are highly penetrant cancer susceptibility genes. The 2015 update, added two more genes related to cancer to the minimum list: *SMAD4* (MIM 600993) and *BMPR1A* (MIM 601299)^1^. The disadvantage of a published minimum list is that it needs constant update, considering that cancer is one of the fields of medicine with the fastest development^11^.

In this context, we aimed to retrospectively investigate germline variants in 94 genes, causally implicated in oncogenesis, in patients referred for NGS testing for various rare genetic diseases, reevaluate them, and discuss the utility of reporting different classes of pathogenicity to the referring physician, families and patients.

## Material and Method

The referrals for genetic testing were children presenting disorders with intellectual disability with or without congenital anomalies, neuro-muscular diseases, inborn errors of metabolism, hematologic diseases, immunodeficiencies, genodermatoses, skeletal dysplasia or endocrine diseases. The request for constitutional TruSightOne panel (Illumina) testing was made at the discretion of the referring clinical geneticist in the Timis Regional Centre of Medical Genetics, affiliated with “Louis Turcanu” Emergency Hospital for Children.

### Sequencing analysis

Libraries were generated according to manufacturer’s protocols using TruSightOne kits (Illumina Inc., San Diego, CA, USA) in the Center of Genomic Medicine from the University of Medicine and Pharmacy “Victor Babes” Timisoara. Targeted DNA sequencing was performed on TruSightOne library, targeting 4813 genes, and sequenced on a MiSeq(Illumina).

Only SNVs that were reported to patients and relevant for their referral were confirmed by Sanger sequencing, including all low-quality variants (GATK quality score below 500). Some variants were confirmed among familial segregation analysis or parental carrier.

### Bioinformatics

The secondary analysis used the Illumina MiSeq Reporter 2.6.2.3 platform, incorporating FASTQ alignment (using Burrows-Wheeler Align version 0.7.9a-isis-1.0.0)^12^, and variant extraction (using SAMtools 0.1.18^13^ and GATK 1.6–23). Sequences were mapped to GRCh37 (“hg19”), retaining reads with a median quality score genotype quality (GQ) greater than 30, variant frequency greater than 20%, variant depth greater than 20 and strand bias less than −10. The VFC annotation was performed using ANNOVAR^14^. The gene-based annotation used the refGene dataset from 2017.06.01, in silico predictors (SIFT, PolyPhen2, CADD, MutationTaster, MutationAsessor, etc) obtained from the dbnsfp3.5.a dataset provided by ANNOVAR (detailed in supplementary information 2), the CLINVAR used the dataset from 2019.10.03. Variant frequency datasets were gnomAD version 2.0.1^15^. Additionally, allele frequency from in-house variant database, were calculated to exclude platform-specific false positive calls as well as to compare phenotypes of rare variant carriers. In silico prediction relied on Combined Annotation Dependent Depletion (CADD) scores^16^ as a tool that integrates multiple annotations such as conversion metrics, functional genomic data, transcript information and protein level scores, and computes a score that indicates the variant effect.

### Filtering strategy

Variants were subject to filtering, which excluded variants with allele frequency in gnomAD(all) higher than 1%^17^, or higher than 5% in house frequency. Also, variants were excluded if reported as benign and likely benign in ClinVar or InterVar, and those with CADDphred score below 20. Higher CADD scores indicate that a variant is more likely to have deleterious effects. A scaled score of 10 or greater indicates a raw score in the top 10% of all possible reference genome SNVs, and a score of 20 or greater indicates a raw score in the top 1%^16^. Synonyms variants and those with a CADDphred score lower than 20 were excluded, unless reported as pathogenic or likely pathogenic in ClinVar. Data was checked for compound heterozygosity. All DNA sequencing results were manually reviewed by two clinical geneticists to prioritize variants and subsequent reporting of consensus variants.

### Data interpretation and reporting

For clinical use the patient variants were classified according to the American College of Medical Genetics and Genomics (ACMG) guidelines^18^ in relation to patient’s phenotype. Pathogenic, likely pathogenic and variants of uncertain significance (VOUS) related to the phenotype were reported for clinical use. Unsolicited findings (outside the ACMG minimum recommended list) were reported to patients, if explicitly described in the consent form and considered of clinical relevance.

### Selection of relevant cancer susceptibility genes

We used the COSMIC (Catalogue Of Somatic Mutations In Cancer) database, downloaded in January 2019 (https://cancer.sanger.ac.uk), to select genes causally implicated in oncogenesis. Out of 723 curated genes, 102 were considered to have a germline effect. Of these we excluded 6 that were not in the TruSightOne panel (*FAT1* MIM 600976, *LMO1* MIM 186921, *LZTR1* MIM 600574, *POLE* MIM174762, *POLQ* MIM 604419, *SPOP* MIM602650) and 14 genes as having a clear role in somatic mutations, but less as germline mutations (*APOBEC3B* MIM 607110, *AR* MIM 313700, *BUB1B* MIM 602860, *CXCR4* MIM 162643, *CYLD* MIM 605018, *ERBB4* MIM 600543, *MPL* MIM 159530, *PTPN13* MIM 400041, *SBDS* MIM 607444, *SETBP1* MIM 611060, *STAT3* MIM 102582, *TP63* MIM 603273, *TSHR* MIM 603372, *WRN* MIM 604611). We added other 12 genes that were shown in studies^19–22^ and MIM database (https://www.omim.org/) to be associated with germline variants causally involved in cancer (*EPCAM, FANCB, FANCL, GALNT12, HOXB13, MITF, PHB, RAD50, RAD51A, RAD51C, RAD51D, RAD54L*). Finally, 94 selected genes of interest, with germline variants that could impact cancer susceptibility are listed in Table 1. Penetrance was reviewed from literature^19–22^ and classified as high, moderate and low, however the penetrance remained unknown for several genes.

**Table 1:** Selected genes with high/moderate and low or unknown cancer predisposition in alphabetical order, autosomal dominant first, followed by autosomal recessive and X-linked transmission.

Gene name	Location hg19	Gene MIM no.	Tumour Types(Germline)	Cancer Syndrome	Inheri-tance	ACMG recomm.
**High risk genes with established predictions with surveillance recommendations**						
*APC*	5q22.2	611731	colorectal, pancreatic, desmoid, hepatoblastoma, glioma, other CNS	adenomatous polyposis coli; Turcot syndrome	AD	yes
*BMPR1A*	10q23.2	601299	gastrointestinal polyps	juvenile polyposis	AD	yes
*BRCA1*	17q21.31	113705	breast, ovarian	hereditary breast/ovarian cancer	AD	yes
*BRCA2*	13q13.1	600185	breast, ovarian, pancreatic, leukaemia	hereditary breast/ovarian cancer	AD	yes
*CDH1*	16q22.1	192090	gastric	familial gastric carcinoma	AD	no
*CDK4*	12q14.1	123829	melanoma	familial malignant melanoma	AD	no
*CDKN2A*	9p21.3	600160	melanoma, pancreatic	familial malignant melanoma	AD	no
*EPCAM*	2p21	185535	colorectal	Colorectal cancer, hereditary nonpolyposis, type 8	AD	no
*FH*	1q43	136850	leiomyomatosis, renal	hereditary leiomyomatosis and renal cell cancer	AD	no
*FLCN*	17p11.2	607273	renal, fibrofolliculomas, trichodiscomas	Birt-Hogg-Dube syndrome	AD	no
*MEN1*	11q13.1	613733	parathyroid adenoma, pituitary adenoma, pancreatic islet cell, carcinoid	multiple endocrine neoplasia type 1	AD	yes
*MLH1*	3p22.2	120436	colorectal, endometrial, ovarian, central nervous system	hereditary non-polyposis colorectal cancer, Turcot syndrome	AD	yes
*MSH2*	2p21-p16	609309	colorectal, endometrial, ovarian	hereditary non-polyposis colorectal cancer	AD	yes
*MSH6*	2p16.3	600678	colorectal, endometrial, ovarian	hereditary non-polyposis colorectal cancer	AD	yes
*MUTYH*	1p34.1	604933	colorectal	adenomatous polyposis coli	AR	yes
*NF1*	17q11.2	613113	neurofibroma, glioma	neurofibromatosis type 1	AD	no
*NF2*	22q12.2	607379	meningioma, acoustic neuroma	neurofibromatosis type 2	AD	yes
*PMS2*	7p22.1	600259	colorectal, endometrial, ovarian, medulloblastoma, glioma	hereditary non-polyposis colorectal cancer, Turcot syndrome	AD	yes
*PTCH1*	1p34.1	603673	skin basal cell, medulloblastoma	nevoid basal cell carcinoma syndrome	AD	no
*PTEN*	10q23.31	601728	harmartoma, glioma, prostate, endometrial	Cowden syndrome, Bannayan-Riley-Ruvalcaba syndrome	AD	yes
*RB1*	13q14.2	614041	retinoblastoma, sarcoma, breast, small cell lung carcinoma	familial retinoblastoma	AD	yes
*RET*	10q11.21	164761	medullary thyroid, papillary thyroid, pheochromocytoma	multiple endocrine neoplasia 2A/2B	AD	yes
*SDHB*	1p36.13	185470	paraganglioma, pheochromocytoma	familial paraganglioma	AD	yes
*SDHD*	11q23.1	602690	paraganglioma, pheochromocytoma	familial paraganglioma	AD	yes
*SDHAF2*	11q12.2	613019	paraganglioma	familial paraganglioma	AD	yes
*SDHC*	1q23.3	602413	paraganglioma, pheochromocytoma	familial paraganglioma	AD	yes
*SMAD4*	18q21.2	600993	gastrointestinal polyp	juvenile polyposis	AD	yes
*STK11*	19p13.3	602216	jejunal hamartoma, ovarian, testicular, pancreatic	Peutz-Jeghers syndrome	AD	yes
*TGFBR2*	3p24.1	190182	colorectal	Hereditary Nonpolyposis Colorectal Cancer type 6	AD	yes
*TP53*	17p13.1	191170	breast, sarcoma, adrenocortical carcinoma, glioma, multiple other tumour types	Li-Fraumeni syndrome	AD	yes
*TSC1*	9q34.13	605284	hamartoma, renal cell carcinoma, tuberous sclerosis tuber	Tuberous sclerosis 1	AD	yes
*TSC2*	16p13.3	191092	hamartoma, renal cell carcinoma, tuberous sclerosis tuber	Tuberous sclerosis 2	AD	yes
*VHL*	3p25.3	608537	renal, haemangioma, pheochromocytoma	Von Hippel-Lindau syndrome	AD	yes
*WT1*	11p13	607102	Wilms tumour	Denys-Drash syndrome, Frasier syndrome, familial Wilms tumour	AD	yes
*FANCA*	16q24.3	607139	Acute myeloid leukemia, leukaemia	Fanconi anaemia complementation group A	AR	no
*FANCB*	Xp22.2	300515	Acute myeloid leukemia, leukaemia	Fanconi anemia, complementation group B	XLR	no
*FANCC*	9q22.32	613899	Acute myeloid leukemia, leukaemia	Fanconi anaemia complementation group C	AR	no
*FANCD2*	3p25.3	613984	Acute myeloid leukemia, leukaemia	Fanconi anaemia complementation group D2	AR	no
*FANCE*	6p21.31	613976	Acute myeloid leukemia, leukaemia	Fanconi anaemia complementation group E	AR	no
*FANCF*	11p14.3	613897	Acute myeloid leukemia, leukaemia	Fanconi anaemia complementation group F	AR	no
*FANCG*	9p13.3	602956	Acute myeloid leukemia, leukaemia	Fanconi anaemia complementation group G	AR	no
*FANCL*	2p16.1	608111	Acute myeloid leukemia, leukaemia	Fanconi anemia, complementation group L	AR	no
*EGFR*	7p11.2	131550	Non-small-cell lung carcinoma	familial lung cancer	AR	no
*ERCC2*	19q13.32	126340	skin basal cell, skin squamous cell, melanoma	Xeroderma pigmentosum, group D	AR	no
*ERCC3*	2q14.3	133510	skin basal cell, skin squamous cell, melanoma	Xeroderma pigmentosum, group B	AR	no
*ERCC4*	16p13.12	133520	skin basal cell, skin squamous cell, melanoma	Xeroderma pigmentosum, group F	AR	no
*ERCC5*	13q33.1	133530	skin basal cell, skin squamous cell, melanoma	Xeroderma pigmentosum, group G	AR	no
*EXT1*	8q24.11	608177	exostoses, osteosarcoma	multiple exostoses type 1	AR	no
*NBN*	8q21.3	602667	Non-Hodgkin lymphoma, glioma, medulloblastoma, rhabdomyosarcoma	Nijmegen breakage syndrome	AR	no
***Moderate/increased risk genes that are acknowledged guidelines screening and risk reduction measures in accordance with family history***						
*ALK*	2p23.2-p23.1	105590	neuroblastoma	familial neuroblastoma	AD	no
*ATM*	11q22.3	607585	leukaemia, lymphoma, medulloblastoma, glioma	ataxia-telangiectasia	AD	no
*BAP1*	3p21.1	603089	mesothelioma, uveal melanoma	Tumor predisposition syndrome	AD	no
*BRIP1*	17q23.2	605882	AML, leukaemia, breast	Fanconi anaemia J, breast cancer susceptiblity	AD	no
*CHEK2*	22q12.1	604373	breast	familial breast cancer	AD	no
*PALB2*	16p12.2	610355	Wilms tumour, medulloblastoma, AML, breast	Fanconi anaemia N, breast cancer susceptibility	AD	no
*RAD51C*	17q22	602774	Breast,ovarian cancer	Breast-ovarian cancer, familial, susceptibility to, 3	AD	no
*RAD51D*	17q12	602954	Breast,ovarian cancer	Breast-ovarian cancer, familial, susceptibility to, 4	AD	no
***Genes that have moderate or high risk based on published studies but pending professional recommendations regarding surveillance and risk reduction strategies***						
*ATR*	3q23	601215	oropharyngeal	familial cutaneous telangiectasia and cancer syndrome, Seckel Syndrome	AD	no
*AXIN2*	17q24.1	604025	colorectal carcinoma	oligodontia-colorectal cancer syndrome	AD	no
*BARD1*	2q35	601593	ovarian cancer, breast cancer, endometrioid cancer		AD	no
*BLM*	15q26.1	604610	leukaemia, lymphoma, skin squamous cell, other tumour types	Bloom syndrome	AD	no
*CDC73*	1q31.2	607393	parathyroid adenoma, multiple ossifying jaw fibroma	hyperparathyroidism-jaw tumour syndrome	AD	no
*CDKN1B*	12p13.1	600778	pituitary, parathyroid	multiple endocrine neoplasia type IV	AD	no
*DICER1*	14q32.13	606241	pleuropulmonary blastoma	familial pleuropulmonary blastoma or DICER1 syndrome	AD	no
*EXT2*	11p11.2	608210	exostoses, osteosarcoma	multiple exostoses type 2	AD	no
*GALNT12*	9q22.33	610290	colorectal	Colorectal cancer, susceptibility to, 1	AD	no
*HNF1A*	12q24.31	142410	hepatic adenoma, hepatocellular carcinoma	familial hepatic adenoma	AD	no
*HOXB13*	17q21-q22	604607	prostate	Prostate cancer, hereditary, 9	AD	no
*HRAS*	11p15.5	190020	rhabdomyosarcoma, ganglioneuroblastoma, bladder	Costello syndrome	AD	no
*KDR*	4q12	191306	melanoma	Hemangioma, capillary infantile, susceptibility to	AD	no
*KIT*	4q12	164920	gastrointestinal, epithelioma	familial gastrointestinal stromal tumour	AD	no
*MAX*	14q23.3	154950	pheochromocytoma	Pheochromocytoma, susceptibility to	AD	no
*MITF*	3p13	156845	melanoma	Melanoma, cutaneous malignant, susceptibility to, 8	AD	no
*PDGFRA*	4q12	173490	gastrointestinal stromal tumour	familial gastrointestinal stromal tumour	AD	no
*PHB*	17q21.33	176705	Breast cancer	{Breast cancer, susceptibility to}	AD	no
*PHOX2B*	4p13	603851	neuroblastoma	familial neuroblastoma	AD	no
*POLD1*	19q13.33	174761	colorectal	Lynch syndrome	AD	no
*PRF1*	10q22.1	170280	various leukaemia, lymphoma		AD	no
*PRKAR1A*	17q24.2	188830	myxoma, endocrine, papillary thyroid	Carney complex	AD	no
*RAD50*	5q31.1	604040	breast cancer	Nijmegen breakage syndrome-like disorder	AR	no
*RAD51A*	15q15.1	179617	breast cancer	?Fanconi anemia, complementation group R	AD	no
*RAD54L*	1p34.1	603615	breast cancer	Breast cancer, invasive ductal	AD	no
*SDHA*	5p15.33	600857	paraganglioma	paragangliomas-5 (PGL5)	AD	no
*SMARCB1*	22q11.23	601607	malignant rhabdoid	rhabdoid predisposition syndrome	AD	no
*SMARCE1*	17q21.2	603111	meningioma	Meningioma, familial, susceptibility to	AD	no
*TERT*	5p15.33	187270	melanoma	Melanoma, cutaneous malignant, 9	AD	no
*TMEM127*	2q11.2	613403	pheochromocytoma, renal cell carcinoma	Pheochromocytoma, susceptibility to	AD	no
*DDB2*	11p11.2	600811	skin basal cell, skin squamous cell, melanoma	xeroderma pigmentosum (E)	AR	no
*RECQL4*	8q24.3	603780	osteosarcoma, skin basal cell, skin sqamous cell	Rothmund-Thompson syndrome	AR	no
*XPA*	9q22.33	611153	skin basal cell, skin squamous cell, melanoma	xeroderma pigmentosum (A)	AR	no
*XPC*	3p25.1	613208	skin basal cell, skin squamous cell, melanoma	xeroderma pigmentosum (C)	AR	no
*SUFU*	10q24.32	607035	medulloblastoma	medulloblastoma predisposition	AR, AD	no
*GPC3*	Xq26.2	300037	Wilms tumour	Simpson-Golabi-Behmel syndrome	XLR	no
*WAS*	Xp11.23	300392	lymphoma	Wiskott-Aldrich syndrome	XLR	no

### Ethics

Written informed consent was obtained from all participant’s legal guardians, after the risks and benefits had been explained to the parent, caregiver or patient. The consent offered the possibility to opt yes or no for disclosure of secondary findings, unrelated to the referral condition. Patients could choose if they wished to have their samples and/or data stored for future research, both anonymously or not. Along the 94 selected genes, only, the variants that were reported to families had phenotype data available, all other variants were irreversibly anonymized. All experiments were performed in accordance with relevant guidelines and regulations. University of Medicine and Pharmacy Timisoara Ethics Committee approved this retrospective analysis of partially anonymized genomics data (no 02/22.01.2019).

## Results

From 1034 individuals evaluated between 2015–2018, a number of 145 individuals (12.1%) had NGS solo analysis using the TruSightOne Illumina Panel. Of them, two caregivers opted out of being informed about unsolicited findings in informed consent process. By retrospectively reanalyzing anonymized sequencing data in 94 genes related to cancer, we identified 18250 variants that passed the quality control threshold. After filtering, a total of 126 variants were considered (shown in Table 2 and in Supplementary Table 1 -with full annotation). Of these, six variants came from 5 individuals that had a cancer related syndrome as indication for analysis (highlighted in red in Supplementary Table 1). These were: compound heterozygous variants in *BRCA1* gene, identified as disease causing for Anemia Fanconi like syndrome; homozygous variant in *NBN* gene, was disease-causing for Nijmegen Syndrome; homozygous variant in *FANCC* gene, was disease-causing for Fanconi Anemia group C; homozygous variant in *FANCA*, was disease-causing for Fanconi Anemia group A and heterozygous variant in *BMPR1A* gene was disease-causing for Juvenile polyposis syndrome.

**Table 2:** Candidate variants (HGVS) in cancer-susceptibility genes observed in the cohort after filtering.

Gene name	HGVS nomenclature	Exonic Function	CADD1.4 Phred	gnomAD all freq	ClinVar	InterVar	rs
Diagnostic variants related to the phenotype							
*BRCA1*	NM_007294.3:c.2933dupA p.(Tyr978Ter)	stopgain	.	.	P	.	rs878853292
*BRCA1*	NM_007294.3:c.843_846delCTCA p.(Ser282Tyr)	frameshift deletion	.	.	P	.	rs80357919
*BMPR1A*	NM_004329.2:c.1439 G > T p.(Arg480Leu)	missense	33	.	VOUS	VOUS	rs535109719
*FANCA*	NM_000135.4:c.295 C > T p.(Gln99Ter)	stopgain	36	.		P	rs1057516430
*FANCC*	NM_000136.2:c.37 C > T p.(Gln13Ter)	stopgain	36	.	P/LP	P	rs121917784
*NBN*	NM_002485.4:c.657_661delACAAA p.(Lys219AsnfsTer16)	Frameshift deletion	.	0.00030	P	.	rs587776650
Pathogenic and likely pathogenic variants unrelated to the phenotype (incidental) - reported to patients							
*BRCA2*	NM_000059.3:c.8331 + 1 G > A	-	34		P	.	rs81002837
*PALB2*	NM_024675.3:c.93dupA p.(Leu32ThrfsTer11)	frameshift insertion	.	.	P/LP	.	rs864622498
*RAD50*	NM_005732.3:c.3050 G > A p.(Trp1017Ter)	stopgain	45	.	P	P	.
Pathogenic and likely pathogenic variants NOT reported to patients							
*ATR*	NM_001184.3:c.7273 C > T p.(Arg2425Ter)	stopgain	43	.	.	P	rs1310011888
*BLM*	NM_000057.3:c.1642C > T p.(Gln548Ter)	stopgain	35	0.00040	P/LP	P	rs200389141
*FANCB*	NM_152633.3:c.2254 G > T p.(Glu752Ter)	stopgain	39	0.00010	.	P	
*MUTYH*	NM_001128425.1:c.1437_1439delGGA p.(Glu480del)	Non-frameshift deletion	.	0.0000323	p	.	rs587778541
*XPC*	NM_004628.4:c.1677C > A p.(Tyr559Ter)	stopgain	36	.	P	P	rs767569346
Secondary findings NOT reported to patients -with high likelihood for pathogenicity							
BLM	NM_000057.4:c.3062 A > G p.(Asn1021Ser)	missense	23.1	.	VOUS	VOUS	rs369629509
BRCA1	NM_007294.3:c.2666 C > T p.(Ser889Phe)	missense	18.58	.	Conflicting interpretations	VOUS	rs769712441
BRCA2	NM_000059.3:c.8735 C > T p.(Ala2912Val)	missense	23.7	.	.	VOUS	.
BRCA2	NM_000059.3:c.8320 C > G p.(Leu2774Val)	missense	26.5	.	.	VOUS	.
CHEK2	NM_007194.4:c.482 A > G p.(Glu161Gly)	missense	28.1	.	VOUS	VOUS	rs730881683
DICER1	NM_030621.4:c.3591 C > G p.(Cys1197Trp)	missense	24.6	.	.	VOUS	.
ERCC4	NM_005236.2:c.934 T > G p.(Ser312Ala)	missense	25.9	0.0000646	.	.	rs200596978
MLH1	NM_000249.3:c.41 C > T p.(Thr14Ile)	missense	24.5	.	VOUS	VOUS	rs774363593
RET	NM_020975.6:c.2330 A > G p.(Asn777Ser)	missense	20.6	.	VOUS	VOUS	rs377767415
SDHB	NM_003000.2:c.230 T > A p.(Ile77Asn)	missense	29.6	.	.	VOUS	.
TP53	NM_000546.5:c.665 C > T p.(Pro222Leu)	missense	19.42	0.0000646	VOUS	VOUS	rs146340390

HGVS = Human Genome Variation Society; Freq = frequency; P = Pathogenic; LP = Likely Pathogenic, VOUS = variant of unknown significance.

Pathogenic and likely pathogenic variants reported to patients (shown in Table 2 and in Supplementary Table 1 in red - with full annotation). Three variants identified, in 3 individuals, identified as likely pathogenic, were reported to patients.

The heterozygous variant c.7273 C > T(p.Arg2425Ter) in *PALB2* gene, was reported as likely pathogenic as a risk for breast cancer, in one child presenting with dystonia. Family history was negative in this case. Both parents were invited for genetic counseling, however, only the mother attended the meeting. She did not request family screening for the variant for the time being, but mentioned she will consider it.

The variant c.8331 + 1 G > A in *BRCA2* was reported as likely pathogenic as a risk for breast cancer, in a boy presenting with thrombocytopenia. Family history was also negative. After genetic counseling with both parents, family screening was desired and carried out, demonstrating the variant’s presence in the father and sister of the patient. The potential risks for people with *BRCA2* pathogenic variants, considering the male gender and young age of the girl, were discussed with the family.

The variant c.3050 G > A (p.Trp1017Ter) in *RAD50* in a boy presenting for myopathy. The patient had a sister with Hodgkin Lymphoma that deceased. The family did not desire carrier testing in the family at the time of genetic counseling.

Pathogenic and likely pathogenic variants (secondary findings) NOT reported to patients associated with susceptibility to cancer (shown in Table 2 and in Supplementary Table 1 in pink- with full annotation). One heterozygous variant c.93dupA p.(Leu32Thrfs) in autosomal dominant *ATR* gene (in one patient). One heterozygous variant c.3532 G > A p.(Gly1178Arg) in X linked recessive *FANCB* gene (in one patient). One heterozygous variant c.1642C > T p.(Gln548Ter) in autosomal recessive *BLM* gene (in two patients). Heterozygous variants in *BLM* and *FANCB (in females)* have the potential to be associated with increased susceptibility to cancer.

Additionally, two heterozygous variants in autosomal recessive genes associated with cancer predisposition, were identified in *MUTYH* gene and *XPC* gene (each in 1 patient). Matching compound heterozygous variants were not identified in these patients. Currently there is insufficient evidence of causality for heterozygous variants in these genes in relation to cancer.

Variants of unknown significance with potential for pathogenicity (secondary findings), NOT reported to patients (shown in Table 2 and in Supplementary Table 1 in blue - with full annotation). Eleven variants that met three of the ACMG criteria to classify pathogenic variants were identified in heterozygous state in *BLM, BRCA2, CHEK2, DICER1, ERCC4, MLH1, RET, SDHB, TP53* genes (each in 1 patient), and in *BRCA1* gene (in 2 patients). Except for *ERCC4*, all these genes have autosomal dominant transmission.

Nine patients had an association of VOUS in two different genes while three patients had associations of VOUS in 3 different genes. Two compound heterozygote variants c.679 G > A(p.Ala227Thr) and c.1248 C > A(p.Leu420Met) in *MUTYH* gene, were identified in one patient. Variants were not reported, as they were considered VOUS. Thirty-two VOUS were positioned in intronic, UTR3 and UTR5 regions.

## Discussion

The ACMG 2013 guideline regarding incidental findings in children state that: “results from genetic testing of a child may have implications for the parents and other family members. Health care providers have an obligation to inform parents and the child, when appropriate, about these potential implications”^2^. The reasons provided by the Working Group for these highly debated recommendations were that: “at this moment in the evolution of clinical sequencing, an incidental finding relevant to adult disease that is discovered and reported through clinical sequencing of a child may be the only way in which that variant will come to light for the parent…Tailoring the reporting of such information according to the age of the patient could place an unrealistic burden upon laboratories facing increasing volumes of clinical sequencing”^2^.

Although of great medical interest, the discovery of cancer predisposing genes, can be overwhelming and might affect life quality for the people involved, affecting decisions related to family planning. Cancer development is complex and several factors influence their development: incomplete penetrance and variability of allele expression in cancer predisposing genes, copy number variants, effect of modifier genes, digenic or oligogenic inheritance pattern, age and gender related penetrance, epigenetic alterations, and environmental exposures (lifestyle)^20^. It is extremely important to counsel patients and families, so they understand that penetrance, expressivity and severity can vary tremendously, in and between families.

Granting we can now generate large amounts of sequence data, our ability to accurately interpret this information, is still limited, creating a significant increase in the numbers of VOUS^19^. Possibly the greatest worry is the likelihood of reporting a false positive unsolicited finding to a patient, due to its potential negative impact. A recent study showed that half of the laboratories did not report any unsolicited findings, while others reported only once the variant had been discussed within a board formed by an independent doctor, ethicist and lawyer^5^.

### Likely pathogenic variants reported to patients in our cohort

*BRCA2* variant identified in the patient was previously published and considered predisposing for breast cancer. The gene is included in the minimum gene list of ACMG recommendation. The parents understood this finding might imply an increased susceptibility to cancer and opted to screen the family members for this variant. The finding created some distress, especially in the waiting time before family screening results. After results of tests the family felt relieved, to some extent, that they have a prognosis and a course of action.

The heterozygous variant in *PALB2* identified in our patient was previously published as deleterious in one patient with breast cancer^23^. *PALB2* gene is considered to have an intermediate risk for breast cancer, conferring a 2- to 3-fold increased risk of breast cancer^24^ and is not in the minimum gene list of ACMG recommendation. Nonetheless, is included in many breast cancer panels. Biallelic *PALB2* pathogenic variants were showed to cause Fanconi anemia, similar to *BRCA1* and *BRCA2*^25^. Family history is an important aspect to evaluate in this context, however in a recent study only 5 of 21 *PALB2* mutation carriers had a family history of breast or ovarian cancer. Thus, many patients with pathogenic variants in predisposition genes may not be identified by a family history of cancer^23^. For this patient, only the mother came for counselling, although both parents were invited. The option to test the variant was offered and the mother mentioned it will be considered after discussion with her partner. Notably her main concern was the condition of the child, granting less importance to a cancer predisposing gene.

Pathogenic variants in *RAD50* was shown to be associated with genomic instability assessed by cytogenetic analysis of peripheral blood T-lymphocytes^26^, suggesting an effect for *RAD50* haploinsufficiency on genomic integrity and susceptibility to cancer. A larger, more recent study has characterized the gene with intermediate-risk breast cancer susceptibility^27^. In our patient the presence of this variant was considered possibly relevant for the sister’s phenotype. The family did not wish to continue with variant screening. In counselling, it seemed that discussing the death of the sister was unsettling, suggesting this was a possible cause for their decision.

### Likely pathogenic variants NOT reported to patients in our cohort

The heterozygous variant in *ATR* gene in our cohort was not previously reported as deleterious, however it is a stopgain variant with deleterious in silico predictions, unreported in ExAc or GnomAD. *ATR* gene is not in the minimum gene list of ACMG recommendation, nonetheless, is involved in DNA-replication and repair. Pathogenic autosomal-recessive variants in ATR gene were reported in Seckel syndrome and recently it has translated into an autosomal-dominant inherited disease encompassing oropharyngeal cancer, skin telangiectases, and mild developmental anomalies of the hair, teeth, and nails^28^. In this case the benefit and risk of reporting are similar to that of *PALB2* gene, however there is less evidence of pathogenicity and unidentified penetrance.

*BLM* gene was associated with autosomal recessive Bloom syndrome, while heterozygous status was associated to breast cancer susceptibility in several studies^29^. A metanalysis showed that *BLM* pathogenic variants were associated with a 2 to 5-fold increase in breast cancer^30^. However a longitudinal study showed that the standardized incidence rates for cancer were not higher than expected and thus heterozygous pathogenic variants carriers are not at increased risk for developing cancer^31^. The p.Gln548Ter variant in *BLM* gene was previously identified as a Slavic founder mutation^30^. The nonsense variant in our cohort was identified in 2 individuals. The benefit and risk of reporting to patient are similar to that of *PALB2* gene.

The FANCB gene is not in the minimum ACMG recommended list, however, identifying X linked carries is relevant for future pregnancies. However, germline heterozygous variants in *FANCB* were associated with increased susceptibility for head and neck carcinoma^32,33^.

Reporting unsolicited variants to the family in NGS testing for a rare disease in children, might be the only opportunity to learn about a variant, which could become relevant at reproductive age and later into adult life. The parents could undergo cancer screening, if carrier status was demonstrated. However, the benefit cannot be fully estimated, nor the risk of creating distress when living under Damocles’ sword^34^. Therefore, parents informed decision is crucial. Notably, the parent’s understanding of the possibility to receive such unsettling news, could be difficult to comprehend^35^, considering the child is referred for genetic testing due to a rare disease that is usually already a major health issue. For the 3 cases where we reported unsolicited variants, the benefits and concerns of the families were different. This variability could be associated with educational status and familial context.

In our cohort, 12 individuals (8.2%) presented 11 variants classified as VOUS, however with a high likelihood for pathogenicity, gathering 3 ACMG criteria for pathogenic variants. Reporting such a variant is considered unethical due to the high risk of being false positive^2^. Nonetheless, the possibility that unsolicited variants classified as VOUS will be reanalyzed is unlikely due to the burden it would create for the laboratories.

The two compound heterozygote variants in *MUTYH* gene identified in one patient, could be missed in our current filtering strategy to identify variants causative for a rare disease. This finding raised the issue that a secondary finding (especially in compound heterozygosity) has a high chance of being overlooked. As *MUTYH* gene is included in the minimum gene list of ACMG recommendation, a laboratory adhering to ACMG recommendations should have bioinformatic strategies to identify it.

Almost five percent (7/145) of individuals in our cohort had unsettling NGS findings (6 variants in 6 genes) related to cancer in retrospective analysis. Lower frequencies were reported by a recent study, where 1% of WES samples had reportable secondary findings in the cancer related genes recommended by ACMG^36^. Similarly, prevalence of pathogenic and likely pathogenic variants in 24 ACMG cancer genes in a family-based cancer research cohort was 1,2% and in cancer-free controls it was 0.8%^37^. However, both studies included only 24 gene recommended in the v.2 minimum list, whereas our study had a much-extended list. Consequently, the higher frequency in our study is due to variants in genes with more recent or less evidence for cancer predisposition. In Kim study^37^, the median review time estimated per-variant was 30 min. The authors highlighted how the analysis of secondary findings required database and literature review, which is a time- and labor-intensive process hindered by the difficulty of interpreting conflicting determinations^37^.

This work has led to change of practice in pre-test genetic counselling in our Center, including: (1) information about family history related to cancer and late onset disease to be enclosed in the details sent to the laboratory; and (2) extended explanation related to course of action after possible identification of unsolicited findings (example: the possibility for testing the variant in other members in the family and screening for the relevant conditions in family members identified at risk after family testing). Additionally, the consent process informs about the distress that these variants could create and the fact that cancer predisposing variants may imply a risk and are not equivalent to a diagnosis.

Considering that cancer is one of the fields of medicine with the fastest development^11^, extending the number of genes in secondary analysis, beyond the ones selected in the minimum recommended list, is needed. In this context, ClinGen (clinicalgenome.org) Hereditary Cancer Gene Curation Working Group focuses on curating cancer predisposition genes for their major associated syndromes. The work will provide a rapidly updatable approach, compared to publishing guidelines. This ongoing effort will facilitate more informed utilization of genomic variants in clinical and cancer research.

### Limitations

Our analysis does not include copy number variants (CNV) in cancer genes. In our cohort CNV were assessed. Although several patients also have SNP array, the results were not included in the manuscript. CNV from sequencing data was not assessed in our cohort. Although having many advantages, large sequencing panels still present some disadvantages compared to Sanger sequencing or smaller NGS panels, such as incomplete coverage of some genes or exons. These tests cannot exclude pathogenic variation. However, despite the limitations, NGS efficiently screen for most variants, supporting their clinical use.

As the authors suggest, the ACMG variant interpretation guideline is imperfect for classifying unsolicited findings^4^. It is important to consider the distinction between implicating a variant as pathogenic (causative for a disease) and a variant that may be predicted to be damaging to the protein but not necessarily implicated in a disease^4^. Authors also state that the use of the ACMG guideline may result in a larger proportion of variants being categorized as uncertain significance^4^. Despite its limitations the guideline is comprehensive and is used extensively for diagnostic and unsolicited findings.

One major limitation is lack of cancer family history information, which is a key component in identifying cancer-predisposition variants. However, Zang *et al*.^38^, showed in their cohort, that family history could not predict an underlying predisposition syndrome in most patients. Furthermore, some individuals with cancer, have de novo predisposing variants, whereas others inherit them with incomplete penetrance; where, the family history is likely to be negative.

## Conclusion

In this retrospective study we have identified 126 germline variants, in 94 genes causally implicated in oncogenesis, in patients referred for NGS testing for various rare genetic diseases. Seven individuals in our cohort (4.8%) had unsolicited findings related to cancer. Six pathogenic and likely pathogenic variants were identified in *BRCA2, PALB2* and *RAD50* genes were reported to families, while variants in *BLM* (in two individuals)*, FANCB, ATR* genes were not reported. All consequently raised difficult ethical debate regarding their reporting. As only the *BRCA2* gene was included in the 2015 ACMG minimum recommended list, we underline the need for constant update of this list. More efforts are needed to create an easily updatable consensus in reporting variants in cancer predisposing genes. Additionally, strategies are required to ensure that patients and physicians understand laboratories NGS reporting practices. Also, the consent process needs to inform of both value and risk of additional genetic information.

## Supplementary information

Software details dbnsfp3.5.a.

Annotated variants after filering (n=126).
