# Supplementary material for: Challenges in reporting pathogenic/potentially pathogenic variants in 94 cancer predisposing genes - in pediatric patients screened with NGS panels
Source: Sci Rep. 2020 Jan 14;10:223. doi: 10.1038/s41598-019-57080-9 (PMC6959212; doi:10.1038/s41598-019-57080-9)
Supplement: Supplementary file 1 — Software details dbnsfp3.5.a. [file 41598_2019_57080_MOESM1_ESM.docx]

Supplementary information 2

**Title: Challenges in reporting pathogenic/potentially pathogenic variants in 94 cancer predisposing genes - in pediatric patients screened with NGS panels**

Authors: Adela Chirita-Emandi^1,2*^ MD,PhD, Nicoleta Andreescu^1,2*^ MD,PhD, Cristian G. Zimbru^1,3^ PhD, Paul Tutac^1,2^MD, Smaranda Arghirescu^4,5^ MD,PhD, Margit Serban^5^ MD,PhD, Maria Puiu^1,2^ MD,PhD

1. Center of Genomic Medicine, Medical Genetics Discipline, University of Medicine and Pharmacy “Victor Babes” Timisoara, 300041, Romania
2. Regional Center of Medical Genetics Timis, Clinical Emergency Hospital for Children “Louis Turcanu” Timisoara, 300011, Romania
3. Politehnica University Timisoara Faculty of Automatics and Computer Science, 300006, Romania
4. Pediatric Department, University of Medicine and Pharmacy “Victor Babes” Timisoara, 300041, Romania
5. Onco-hematology department, Clinical Emergency Hospital for Children “Louis Turcanu” Timisoara, 300011, Romania

*Equal contribution

Correspondence to: Dr. Adela Chirita-Emandi, Email: [adela.chirita@umft.ro](mailto:adela.chirita@umft.ro), telephone: 0040724369599, Center of Genomic Medicine, Genetics Discipline, University of Medicine and Pharmacy “Victor Babes” Timisoara, Romania Address P-ta Eftimie Murgu nr. 2; Timisoara, 300041

**Running title: Germline cancer predisposing genes in children**

**Disclosure: The authors declare no conflict of interest**

dbNSFP version 3.5a

Release:

August 6, 2017

Major sources:

Variant determination:

Gencode release 22/Ensembl 79, released March, 2015 (hg38)

Functional predictions:

SIFT ensembl 66, released Jan, 2015 http://provean.jcvi.org/index.php

PROVEAN 1.1 ensembl 66, released Jan, 2015 http://provean.jcvi.org/index.php

Polyphen-2 v2.2.2, released Feb, 2012 http://genetics.bwh.harvard.edu/pph2/

LRT, released November, 2009 http://www.genetics.wustl.edu/jflab/lrt_query.html

MutationTaster 2, data retrieved in 2015 http://www.mutationtaster.org/

MutationAssessor, release 3 http://mutationassessor.org/

FATHMM, v2.3 http://fathmm.biocompute.org.uk

fathmm-MKL, http://fathmm.biocompute.org.uk/fathmmMKL.htm

CADD, v1.3 http://cadd.gs.washington.edu/

VEST, v3.0 http://karchinlab.org/apps/appVest.html

fitCons, v1.01 http://compgen.bscb.cornell.edu/fitCons/

DANN, https://cbcl.ics.uci.edu/public_data/DANN/

MetaSVM and MetaLR, doi: 10.1093/hmg/ddu733

GenoCanyon, v1.0.3 http://genocanyon.med.yale.edu/index.html

Eigen & Eigen PC， v1.1 http://www.columbia.edu/~ii2135/eigen.html

M-CAP, v1.0 http://bejerano.stanford.edu/MCAP/

REVEL, https://sites.google.com/site/revelgenomics/

MutPred, v1.2 http://mutpred.mutdb.org/

Conservation scores:

phyloP100way_vertebrate (hg38) http://hgdownload.soe.ucsc.edu/goldenPath/hg38/phyloP100way/

phyloP20way_mammalian (hg38) http://hgdownload.soe.ucsc.edu/goldenPath/hg38/phyloP20way/

phastCons100way_vertebrate (hg38) http://hgdownload.soe.ucsc.edu/goldenPath/hg38/phastCons100way/

phastCons20way_mammalian (hg38) http://hgdownload.soe.ucsc.edu/goldenPath/hg38/phastCons20way/

GERP++ http://mendel.stanford.edu/SidowLab/downloads/gerp/

SiPhy http://www.broadinstitute.org/mammals/2x/siphy_hg19/

Other variant annotation sources:

Interpro v64 http://www.ebi.ac.uk/interpro/

1000 Genomes project http://www.1000genomes.org/

ESP http://evs.gs.washington.edu/EVS/

dbSNP 150 (hg38) ftp://ftp.ncbi.nih.gov/snp/organisms/human_9606_b150_GRCh38p7/VCF/00-All.vcf.gz

clinvar 20170530 (hg38) ftp://ftp.ncbi.nlm.nih.gov/pub/clinvar/vcf_GRCh38/clinvar_20170530.vcf.gz

ExAC v0.3 http://exac.broadinstitute.org/

UK10K COHORT http://www.uk10k.org/studies/cohorts.html

Ancestral alleles (hg38) ftp://ftp.ensembl.org/pub/release-89/fasta/ancestral_alleles/homo_sapiens_ancestor_GRCh38_e86.tar.gz

Altai Neanderthal genotypes: http://cdna.eva.mpg.de/neandertal/Vindija/VCF/Altai/

Denisova genotypes: http://cdna.eva.mpg.de/neandertal/Vindija/VCF/Denisova/

RSRS http://dx.doi.org/10.1016/j.ajhg.2012.03.002

GTEx v6p https://www.gtexportal.org/home/datasets

Other gene annotation sources:

HGNC, downloaded on August 4, 2017

Uniprot, release 2017_07

IntAct, downloaded on August 4, 2017

GWAS catalog, r2017-05-29

egenetics and GNF/Atlas expression data, downloaded from BioMart on Oct. 1, 2013

BioGRID, version 3.4.151

Haploinsufficiency probability data, from doi:10.1371/journal.pgen.1001154

Recessive probability data, from DOI:10.1126/science.1215040

Residual Variation Intolerance Score (RVIS), v3

Genome-wide haploinsufficiency score (GHIS), from doi: 10.1093/nar/gkv474

ExAC Functional Gene Constraint, from release0.3.1

ExAC CNV gene score, from release0.3.1

GO, downloaded on August 5, 2017

ConsensusPathDB, Release 32

Essential genes, based on doi:10.1371/journal.pgen.1003484

Mouse genes, from Mouse Genome Informatics (MGI), 6.10

Zebrafish genes, from The Zebrafish Information Network (ZFIN), downloaded on August 5, 2017

KEGG pathway, from http://www.openbioinformatics.org/gengen/tutorial_calculate_gsea.html

BioCarta pathway, from http://www.openbioinformatics.org/gengen/tutorial_calculate_gsea.html

GDI, from doi: 10.1073/pnas.1518646112

LoFtool, from DOI:10.1093/bioinformatics/btv602

SORVA, from doi: 10.1101/103218

Files:

dbNSFP3.5a_variant.chr<#> - dbNSFP variant database files by chromosomes

dbNSFP3.5_gene - dbNSFP gene database file

dbNSFP3.5_gene.complete - dbNSFP gene database file with complete interaction columns

dbscSNV1.1.chr<#> - scSNV database v1.1 files by chromosomes

dbNSFP3.5a.readme.txt - this file

search_dbNSFP35a.class - companion Java program for searching dbNSFP3.5a

search_dbNSFP35a.java - the source code of the java program

LICENSE.txt - the license for using the source code

search_dbNSFP35a.readme.pdf - README file for search_dbNSFP34a.class

tryhg19.in - an example input file with hg19 genome positions

tryhg18.in - an example input file with hg18 genome positions

tryhg38.in - an example input file with hg38 genome positions

try.vcf - an example of vcf input file

Description:

The dbNSFP is an integrated database of functional annotations from multiple

sources for the comprehensive collection of human non-synonymous SNPs (nsSNVs).

Its current version includes a total of 83,422,341 nsSNVs and ssSNVs (splice site

SNVs). It compiles prediction scores from 20 prediction algorithms (SIFT, Polyphen2-HDIV,

Polyphen2-HVAR, LRT, MutationTaster2, MutationAssessor, FATHMM, MetaSVM, MetaLR, CADD,

VEST3, PROVEAN, FATHMM-MKL coding, fitCons, DANN, GenoCanyon, Eigen, Eigen-PC， M-CAP, REVEL, MutPred),

6 conservation scores (phyloP100way_vertebrate, phyloP20way_mammal, phastCons100way_vertebrate,

phastCons20way_mammal, GERP++ and SiPhy) and other function annotations.

Since version 2.0, dbNSFP is separated into two parts, dbNSFP_variant and

dbNSFP_gene. As their names indicate, the former focuses on variant annotations

(including prediction scores and conservation scores), and the latter focuses on

gene annotations.

Since version 2.6, dbscSNV is added as an attached database, which includes all

potential human SNVs within splicing consensus regions (−3 to +8 at the 5’ splice site

and −12 to +2 at the 3’ splice site), i.e. scSNVs, and predictions for their potential

of altering splicing.

Since version 3, two branches of dbNSFP are provided: "a" branch is suitable for academic use,

which includes all the resources, and "c" branch is suitable for commercial use, which does not

include Polyphen2, VEST3, REVEL, CADD and DANN.

Columns of dbNSFP_variant:

1 chr: chromosome number

2 pos(1-based): physical position on the chromosome as to hg38 (1-based coordinate).

For mitochondrial SNV, this position refers to the rCRS (GenBank: NC_012920).

3 ref: reference nucleotide allele (as on the + strand)

4 alt: alternative nucleotide allele (as on the + strand)

5 aaref: reference amino acid

"." if the variant is a splicing site SNP (2bp on each end of an intron)

6 aaalt: alternative amino acid

"." if the variant is a splicing site SNP (2bp on each end of an intron)

7 rs_dbSNP150: rs number from dbSNP 150

8 hg19_chr: chromosome as to hg19, "." means missing

9 hg19_pos(1-based): physical position on the chromosome as to hg19 (1-based coordinate).

For mitochondrial SNV, this position refers to a YRI sequence (GenBank: AF347015)

10 hg18_chr: chromosome as to hg18, "." means missing

11 hg18_pos(1-based): physical position on the chromosome as to hg18 (1-based coordinate)

For mitochondrial SNV, this position refers to a YRI sequence (GenBank: AF347015)

12 genename: gene name; if the nsSNV can be assigned to multiple genes, gene names are

separated by ";"

13 cds_strand: coding sequence (CDS) strand (+ or -)

14 refcodon: reference codon

15 codonpos: position on the codon (1, 2 or 3)

16 codon_degeneracy: degenerate type (0, 2 or 3)

17 Ancestral_allele: ancestral allele based on 8 primates EPO.

Ancestral alleles by Ensembl 84. The following comes from its original README file:

ACTG - high-confidence call, ancestral state supported by the other two sequences

actg - low-confidence call, ancestral state supported by one sequence only

N - failure, the ancestral state is not supported by any other sequence

- - the extant species contains an insertion at this position

. - no coverage in the alignment

18 AltaiNeandertal: genotype of a deep sequenced Altai Neanderthal

19 Denisova: genotype of a deep sequenced Denisova

20 Ensembl_geneid: Ensembl gene id

21 Ensembl_transcriptid: Ensembl transcript ids (Multiple entries separated by ";")

22 Ensembl_proteinid: Ensembl protein ids

Multiple entries separated by ";", corresponding to Ensembl_transcriptids

23 aapos: amino acid position as to the protein.

"-1" if the variant is a splicing site SNP (2bp on each end of an intron).

Multiple entries separated by ";", corresponding to Ensembl_proteinid

24 SIFT_score: SIFT score (SIFTori). Scores range from 0 to 1. The smaller the score the

more likely the SNP has damaging effect.

Multiple scores separated by ";", corresponding to Ensembl_proteinid.

25 SIFT_converted_rankscore: SIFTori scores were first converted to SIFTnew=1-SIFTori,

then ranked among all SIFTnew scores in dbNSFP. The rankscore is the ratio of

the rank the SIFTnew score over the total number of SIFTnew scores in dbNSFP.

If there are multiple scores, only the most damaging (largest) rankscore is presented.

The rankscores range from 0.00963 to 0.91219.

26 SIFT_pred: If SIFTori is smaller than 0.05 (rankscore>0.395) the corresponding nsSNV is

predicted as "D(amaging)"; otherwise it is predicted as "T(olerated)".

Multiple predictions separated by ";"

27 Uniprot_acc_Polyphen2: Uniprot accession number provided by Polyphen2.

Multiple entries separated by ";".

28 Uniprot_id_Polyphen2: Uniprot ID numbers corresponding to Uniprot_acc_Polyphen2.

Multiple entries separated by ";".

29 Uniprot_aapos_Polyphen2: amino acid position as to Uniprot_acc_Polyphen2.

Multiple entries separated by ";".

30 Polyphen2_HDIV_score: Polyphen2 score based on HumDiv, i.e. hdiv_prob.

The score ranges from 0 to 1.

Multiple entries separated by ";", corresponding to Uniprot_acc_Polyphen2.

31 Polyphen2_HDIV_rankscore: Polyphen2 HDIV scores were first ranked among all HDIV scores

in dbNSFP. The rankscore is the ratio of the rank the score over the total number of

the scores in dbNSFP. If there are multiple scores, only the most damaging (largest)

rankscore is presented. The scores range from 0.02634 to 0.89865.

32 Polyphen2_HDIV_pred: Polyphen2 prediction based on HumDiv, "D" ("probably damaging",

HDIV score in [0.957,1] or rankscore in [0.52844,0.89865]), "P" ("possibly damaging",

HDIV score in [0.453,0.956] or rankscore in [0.34282,0.52689]) and "B" ("benign",

HDIV score in [0,0.452] or rankscore in [0.02634,0.34268]). Score cutoff for binary

classification is 0.5 for HDIV score or 0.3528 for rankscore, i.e. the prediction is

"neutral" if the HDIV score is smaller than 0.5 (rankscore is smaller than 0.3528),

and "deleterious" if the HDIV score is larger than 0.5 (rankscore is larger than

0.3528). Multiple entries are separated by ";".

33 Polyphen2_HVAR_score: Polyphen2 score based on HumVar, i.e. hvar_prob.

The score ranges from 0 to 1.

Multiple entries separated by ";", corresponding to Uniprot_acc_Polyphen2.

34 Polyphen2_HVAR_rankscore: Polyphen2 HVAR scores were first ranked among all HVAR scores

in dbNSFP. The rankscore is the ratio of the rank the score over the total number of

the scores in dbNSFP. If there are multiple scores, only the most damaging (largest)

rankscore is presented. The scores range from 0.01257 to 0.97092.

35 Polyphen2_HVAR_pred: Polyphen2 prediction based on HumVar, "D" ("probably damaging",

HVAR score in [0.909,1] or rankscore in [0.62797,0.97092]), "P" ("possibly damaging",

HVAR in [0.447,0.908] or rankscore in [0.44195,0.62727]) and "B" ("benign", HVAR

score in [0,0.446] or rankscore in [0.01257,0.44151]). Score cutoff for binary

classification is 0.5 for HVAR score or 0.45833 for rankscore, i.e. the prediction

is "neutral" if the HVAR score is smaller than 0.5 (rankscore is smaller than

0.45833), and "deleterious" if the HVAR score is larger than 0.5 (rankscore is larger

than 0.45833). Multiple entries are separated by ";".

36 LRT_score: The original LRT two-sided p-value (LRTori), ranges from 0 to 1.

37 LRT_converted_rankscore: LRTori scores were first converted as LRTnew=1-LRTori*0.5 if

Omega<1, or LRTnew=LRTori*0.5 if Omega>=1. Then LRTnew scores were ranked among all

LRTnew scores in dbNSFP. The rankscore is the ratio of the rank over the total number

of the scores in dbNSFP. The scores range from 0.00162 to 0.84324.

38 LRT_pred: LRT prediction, D(eleterious), N(eutral) or U(nknown), which is not solely

determined by the score.

39 LRT_Omega: estimated nonsynonymous-to-synonymous-rate ratio (Omega, reported by LRT)

40 MutationTaster_score: MutationTaster p-value (MTori), ranges from 0 to 1.

Multiple scores are separated by ";". Information on corresponding transcript(s) can

be found by querying http://www.mutationtaster.org/ChrPos.html

41 MutationTaster_converted_rankscore: The MTori scores were first converted: if the prediction

is "A" or "D" MTnew=MTori; if the prediction is "N" or "P", MTnew=1-MTori. Then MTnew

scores were ranked among all MTnew scores in dbNSFP. If there are multiple scores of a

SNV, only the largest MTnew was used in ranking. The rankscore is the ratio of the

rank of the score over the total number of MTnew scores in dbNSFP. The scores range

from 0.08979 to 0.81033.

42 MutationTaster_pred: MutationTaster prediction, "A" ("disease_causing_automatic"),

"D" ("disease_causing"), "N" ("polymorphism") or "P" ("polymorphism_automatic"). The

score cutoff between "D" and "N" is 0.5 for MTnew and 0.31713 for the rankscore.

43 MutationTaster_model: MutationTaster prediction models.

44 MutationTaster_AAE: MutationTaster predicted amino acid change.

45 MutationAssessor_UniprotID: Uniprot ID number provided by MutationAssessor.

46 MutationAssessor_variant: AA variant as to MutationAssessor_UniprotID.

47 MutationAssessor_score: MutationAssessor functional impact combined score (MAori). The

score ranges from -5.135 to 6.49 in dbNSFP.

48 MutationAssessor_rankscore: MAori scores were ranked among all MAori scores in dbNSFP.

The rankscore is the ratio of the rank of the score over the total number of MAori

scores in dbNSFP. The scores range from 0 to 1.

49 MutationAssessor_pred: MutationAssessor's functional impact of a variant :

predicted functional, i.e. high ("H") or medium ("M"), or predicted non-functional,

i.e. low ("L") or neutral ("N"). The MAori score cutoffs between "H" and "M",

"M" and "L", and "L" and "N", are 3.5, 1.935 and 0.8, respectively. The rankscore cutoffs

between "H" and "M", "M" and "L", and "L" and "N", are 0.92922, 0.51944 and 0.19719,

respectively.

50 FATHMM_score: FATHMM default score (weighted for human inherited-disease mutations with

Disease Ontology) (FATHMMori). Scores range from -16.13 to 10.64. The smaller the score

the more likely the SNP has damaging effect.

Multiple scores separated by ";", corresponding to Ensembl_proteinid.

51 FATHMM_converted_rankscore: FATHMMori scores were first converted to

FATHMMnew=1-(FATHMMori+16.13)/26.77, then ranked among all FATHMMnew scores in dbNSFP.

The rankscore is the ratio of the rank of the score over the total number of FATHMMnew

scores in dbNSFP. If there are multiple scores, only the most damaging (largest)

rankscore is presented. The scores range from 0 to 1.

52 FATHMM_pred: If a FATHMMori score is <=-1.5 (or rankscore >=0.81332) the corresponding nsSNV

is predicted as "D(AMAGING)"; otherwise it is predicted as "T(OLERATED)".

Multiple predictions separated by ";", corresponding to Ensembl_proteinid.

53 PROVEAN_score: PROVEAN score (PROVEANori). Scores range from -14 to 14. The smaller the score

the more likely the SNP has damaging effect.

Multiple scores separated by ";", corresponding to Ensembl_proteinid.

54 PROVEAN_converted_rankscore: PROVEANori were first converted to PROVEANnew=1-(PROVEANori+14)/28,

then ranked among all PROVEANnew scores in dbNSFP. The rankscore is the ratio of

the rank the PROVEANnew score over the total number of PROVEANnew scores in dbNSFP.

If there are multiple scores, only the most damaging (largest) rankscore is presented.

The scores range from 0 to 1.

55 PROVEAN_pred: If PROVEANori <= -2.5 (rankscore>=0.543) the corresponding nsSNV is

predicted as "D(amaging)"; otherwise it is predicted as "N(eutral)".

Multiple predictions separated by ";", corresponding to Ensembl_proteinid.

56 Transcript_id_VEST3: Transcript id provided by VEST3.

57 Transcript_var_VEST3: amino acid change as to Transcript_id_VEST3.

58 VEST3_score: VEST 3.0 score. Score ranges from 0 to 1. The larger the score the more likely

the mutation may cause functional change.

Multiple scores separated by ";", corresponding to Transcript_id_VEST3.

Please note this score is free for non-commercial use. For more details please refer to

http://wiki.chasmsoftware.org/index.php/SoftwareLicense. Commercial users should contact

the Johns Hopkins Technology Transfer office.

59 VEST3_rankscore: VEST3 scores were ranked among all VEST3 scores in dbNSFP.

The rankscore is the ratio of the rank of the score over the total number of VEST3

scores in dbNSFP. In case there are multiple scores for the same variant, the largest

score (most damaging) is presented. The scores range from 0 to 1.

Please note VEST score is free for non-commercial use. For more details please refer to

http://wiki.chasmsoftware.org/index.php/SoftwareLicense. Commercial users should contact

the Johns Hopkins Technology Transfer office.

60 MetaSVM_score: Our support vector machine (SVM) based ensemble prediction score, which

incorporated 10 scores (SIFT, PolyPhen-2 HDIV, PolyPhen-2 HVAR, GERP++, MutationTaster,

Mutation Assessor, FATHMM, LRT, SiPhy, PhyloP) and the maximum frequency observed in

the 1000 genomes populations. Larger value means the SNV is more likely to be damaging.

Scores range from -2 to 3 in dbNSFP.

61 MetaSVM_rankscore: MetaSVM scores were ranked among all MetaSVM scores in dbNSFP.

The rankscore is the ratio of the rank of the score over the total number of MetaSVM

scores in dbNSFP. The scores range from 0 to 1.

62 MetaSVM_pred: Prediction of our SVM based ensemble prediction score,"T(olerated)" or

"D(amaging)". The score cutoff between "D" and "T" is 0. The rankscore cutoff between

"D" and "T" is 0.82268.

63 MetaLR_score: Our logistic regression (LR) based ensemble prediction score, which

incorporated 10 scores (SIFT, PolyPhen-2 HDIV, PolyPhen-2 HVAR, GERP++, MutationTaster,

Mutation Assessor, FATHMM, LRT, SiPhy, PhyloP) and the maximum frequency observed in

the 1000 genomes populations. Larger value means the SNV is more likely to be damaging.

Scores range from 0 to 1.

64 MetaLR_rankscore: MetaLR scores were ranked among all MetaLR scores in dbNSFP. The rankscore

is the ratio of the rank of the score over the total number of MetaLR scores in dbNSFP.

The scores range from 0 to 1.

65 MetaLR_pred: Prediction of our MetaLR based ensemble prediction score,"T(olerated)" or

"D(amaging)". The score cutoff between "D" and "T" is 0.5. The rankscore cutoff between

"D" and "T" is 0.81113.

66 Reliability_index: Number of observed component scores (except the maximum frequency in

the 1000 genomes populations) for MetaSVM and MetaLR. Ranges from 1 to 10. As MetaSVM

and MetaLR scores are calculated based on imputed data, the less missing component

scores, the higher the reliability of the scores and predictions.

67 M-CAP_score: M-CAP score (details in DOI: 10.1038/ng.3703). Scores range from 0 to 1. The larger

the score the more likely the SNP has damaging effect.

68 M-CAP_rankscore: M-CAP scores were ranked among all M-CAP scores in dbNSFP. The rankscore is

the ratio of the rank of the score over the total number of M-CAP scores in dbNSFP.

69 M-CAP_pred: Prediction of M-CAP score based on the authors' recommendation, "T(olerated)" or

"D(amaging)". The score cutoff between "D" and "T" is 0.025.

70 REVEL_score: REVEL is an ensemble score based on 13 individual scores for predicting the

pathogenicity of missense variants. Scores range from 0 to 1. The larger the score the more

likely the SNP has damaging effect. "REVEL scores are freely available for non-commercial use.

For other uses, please contact Weiva Sieh" (weiva.sieh@mssm.edu)

71 REVEL_rankscore: REVEL scores were ranked among all REVEL scores in dbNSFP. The rankscore is

the ratio of the rank of the score over the total number of REVEL scores in dbNSFP.

72 MutPred_score: General MutPred score. Scores range from 0 to 1. The larger the score the more

likely the SNP has damaging effect.

73 MutPred_rankscore: MutPred scores were ranked among all MutPred scores in dbNSFP. The rankscore is

the ratio of the rank of the score over the total number of MutPred scores in dbNSFP.

74 MutPred_protID: UniProt accession or Ensembl transcript ID used for MutPred_score calculation.

75 MutPred_AAchange: Amino acid change used for MutPred_score calculation.

76 MutPred_Top5features: Top 5 features (molecular mechanisms of disease) as predicted by MutPred with

p values. MutPred_score > 0.5 and p < 0.05 are referred to as actionable hypotheses.

MutPred_score > 0.75 and p < 0.05 are referred to as confident hypotheses.

MutPred_score > 0.75 and p < 0.01 are referred to as very confident hypotheses.

77 CADD_raw: CADD raw score for functional prediction of a SNP. Please refer to Kircher et al.

(2014) Nature Genetics 46(3):310-5 for details. The larger the score the more likely

the SNP has damaging effect. Scores range from -7.535037 to 35.788538 in dbNSFP.

Please note the following copyright statement for CADD:

"CADD scores (http://cadd.gs.washington.edu/) are Copyright 2013 University of

Washington and Hudson-Alpha Institute for Biotechnology (all rights reserved) but are

freely available for all academic, non-commercial applications. For commercial

licensing information contact Jennifer McCullar (mccullaj@uw.edu)."

78 CADD_raw_rankscore: CADD raw scores were ranked among all CADD raw scores in dbNSFP. The

rankscore is the ratio of the rank of the score over the total number of CADD

raw scores in dbNSFP. Please note the following copyright statement for CADD: "CADD

scores (http://cadd.gs.washington.edu/) are Copyright 2013 University of Washington

and Hudson-Alpha Institute for Biotechnology (all rights reserved) but are freely

available for all academic, non-commercial applications. For commercial licensing

information contact Jennifer McCullar (mccullaj@uw.edu)."

79 CADD_phred: CADD phred-like score. This is phred-like rank score based on whole genome

CADD raw scores. Please refer to Kircher et al. (2014) Nature Genetics 46(3):310-5

for details. The larger the score the more likely the SNP has damaging effect.

Please note the following copyright statement for CADD: "CADD scores

(http://cadd.gs.washington.edu/) are Copyright 2013 University of Washington and

Hudson-Alpha Institute for Biotechnology (all rights reserved) but are freely

available for all academic, non-commercial applications. For commercial licensing

information contact Jennifer McCullar (mccullaj@uw.edu)."

80 DANN_score: DANN is a functional prediction score retrained based on the training data

of CADD using deep neural network. Scores range from 0 to 1. A larger number indicate

a higher probability to be damaging. More information of this score can be found in

doi: 10.1093/bioinformatics/btu703. For commercial application of DANN, please contact

Daniel Quang (dxquang@uci.edu)

81 DANN_rankscore: DANN scores were ranked among all DANN scores in dbNSFP. The rankscore is

the ratio of the rank of the score over the total number of DANN scores in dbNSFP.

82 fathmm-MKL_coding_score: fathmm-MKL p-values. Scores range from 0 to 1. SNVs with scores >0.5

are predicted to be deleterious, and those <0.5 are predicted to be neutral or benign.

Scores close to 0 or 1 are with the highest-confidence. Coding scores are trained using 10

groups of features. More details of the score can be found in

doi: 10.1093/bioinformatics/btv009.

83 fathmm-MKL_coding_rankscore: fathmm-MKL coding scores were ranked among all fathmm-MKL coding

scores in dbNSFP. The rankscore is the ratio of the rank of the score over the total number

of fathmm-MKL coding scores in dbNSFP.

84 fathmm-MKL_coding_pred: If a fathmm-MKL_coding_score is >0.5 (or rankscore >0.28317)

the corresponding nsSNV is predicted as "D(AMAGING)"; otherwise it is predicted as "N(EUTRAL)".

85 fathmm-MKL_coding_group: the groups of features (labeled A-J) used to obtained the score. More

details can be found in doi: 10.1093/bioinformatics/btv009.

86 Eigen_coding_or_noncoding: Whether Eigen-raw and Eigen-phred scores are based on coding model

or noncoding model.

87 Eigen-raw: Eigen score for coding SNVs. A functional prediction score based on conservation,

allele frequencies, and deleteriousness prediction using an unsupervised learning method

(doi: 10.1038/ng.3477).

88 Eigen-phred: Eigen score in phred scale.

89 Eigen-PC-raw: Eigen PC score for genome-wide SNVs. A functional prediction score based on

conservation, allele frequencies, deleteriousness prediction (for missense SNVs) and

epigenomic signals (for synonymous and non-coding SNVs) using an unsupervised learning

method (doi: 10.1038/ng.3477).

90 Eigen-PC-phred: Eigen PC score in phred scale.

91 Eigen-PC-raw_rankscore: Eigen-PC-raw scores were ranked among all Eigen-PC-raw scores in

dbNSFP. The rankscore is the ratio of the rank of the score over the total number

of Eigen-PC-raw scores in dbNSFP.

92 GenoCanyon_score: A functional prediction score based on conservation and biochemical

annotations using an unsupervised statistical learning. (doi:10.1038/srep10576)

93 GenoCanyon_score_rankscore: GenoCanyon_score scores were ranked among all integrated fitCons

scores in dbNSFP. The rankscore is the ratio of the rank of the score over the total number

of GenoCanyon_score scores in dbNSFP.

94 integrated_fitCons_score: fitCons score predicts the fraction of genomic positions belonging to

a specific function class (defined by epigenomic "fingerprint") that are under selective

pressure. Scores range from 0 to 1, with a larger score indicating a higher proportion of

nucleic sites of the functional class the genomic position belong to are under selective

pressure, therefore more likely to be functional important. Integrated (i6) scores are

integrated across three cell types (GM12878, H1-hESC and HUVEC). More details can be found

in doi:10.1038/ng.3196.

95 integrated_fitCons_rankscore: integrated fitCons scores were ranked among all integrated fitCons

scores in dbNSFP. The rankscore is the ratio of the rank of the score over the total number

of integrated fitCons scores in dbNSFP.

96 integrated_confidence_value: 0 - highly significant scores (approx. p<.003); 1 - significant scores

(approx. p<.05); 2 - informative scores (approx. p<.25); 3 - other scores (approx. p>=.25).

97 GM12878_fitCons_score: fitCons score predicts the fraction of genomic positions belonging to

a specific function class (defined by epigenomic "fingerprint") that are under selective

pressure. Scores range from 0 to 1, with a larger score indicating a higher proportion of

nucleic sites of the functional class the genomic position belong to are under selective

pressure, therefore more likely to be functional important. GM12878 fitCons scores are

based on cell type GM12878. More details can be found in doi:10.1038/ng.3196.

98 GM12878_fitCons_rankscore: GM12878 fitCons scores were ranked among all GM12878 fitCons

scores in dbNSFP. The rankscore is the ratio of the rank of the score over the total number

of GM12878 fitCons scores in dbNSFP.

99 GM12878_confidence_value: 0 - highly significant scores (approx. p<.003); 1 - significant scores

(approx. p<.05); 2 - informative scores (approx. p<.25); 3 - other scores (approx. p>=.25).

100 H1-hESC_fitCons_score: fitCons score predicts the fraction of genomic positions belonging to

a specific function class (defined by epigenomic "fingerprint") that are under selective

pressure. Scores range from 0 to 1, with a larger score indicating a higher proportion of

nucleic sites of the functional class the genomic position belong to are under selective

pressure, therefore more likely to be functional important. GM12878 fitCons scores are

based on cell type H1-hESC. More details can be found in doi:10.1038/ng.3196.

101 H1-hESC_fitCons_rankscore: H1-hESC fitCons scores were ranked among all H1-hESC fitCons

scores in dbNSFP. The rankscore is the ratio of the rank of the score over the total number

of H1-hESC fitCons scores in dbNSFP.

102 H1-hESC_confidence_value: 0 - highly significant scores (approx. p<.003); 1 - significant scores

(approx. p<.05); 2 - informative scores (approx. p<.25); 3 - other scores (approx. p>=.25).

103 HUVEC_fitCons_score: fitCons score predicts the fraction of genomic positions belonging to

a specific function class (defined by epigenomic "fingerprint") that are under selective

pressure. Scores range from 0 to 1, with a larger score indicating a higher proportion of

nucleic sites of the functional class the genomic position belong to are under selective

pressure, therefore more likely to be functional important. GM12878 fitCons scores are

based on cell type HUVEC. More details can be found in doi:10.1038/ng.3196.

104 HUVEC_fitCons_rankscore: HUVEC fitCons scores were ranked among all HUVEC fitCons

scores in dbNSFP. The rankscore is the ratio of the rank of the score over the total number

of HUVEC fitCons scores in dbNSFP.

105 HUVEC_confidence_value: 0 - highly significant scores (approx. p<.003); 1 - significant scores

(approx. p<.05); 2 - informative scores (approx. p<.25); 3 - other scores (approx. p>=.25).

106 GERP++_NR: GERP++ neutral rate

107 GERP++_RS: GERP++ RS score, the larger the score, the more conserved the site. Scores range from

-12.3 to 6.17.

108 GERP++_RS_rankscore: GERP++ RS scores were ranked among all GERP++ RS scores in dbNSFP.

The rankscore is the ratio of the rank of the score over the total number of GERP++ RS

scores in dbNSFP.

109 phyloP100way_vertebrate: phyloP (phylogenetic p-values) conservation score based on the

multiple alignments of 100 vertebrate genomes (including human). The larger the score,

the more conserved the site. Scores range from -20.0 to 10.003 in dbNSFP.

110 phyloP100way_vertebrate_rankscore: phyloP100way_vertebrate scores were ranked among all

phyloP100way_vertebrate scores in dbNSFP. The rankscore is the ratio of the rank of the

score over the total number of phyloP100way_vertebrate scores in dbNSFP.

111 phyloP20way_mammalian: phyloP (phylogenetic p-values) conservation score based on the

multiple alignments of 20 mammalian genomes (including human). The larger the score,

the more conserved the site. Scores range from -13.282 to 1.199 in dbNSFP.

112 phyloP20way_mammalian_rankscore: phyloP20way_mammalian scores were ranked among all

phyloP20way_mammalian scores in dbNSFP. The rankscore is the ratio of the rank of the

score over the total number of phyloP20way_mammalian scores in dbNSFP.

113 phastCons100way_vertebrate: phastCons conservation score based on the multiple alignments

of 100 vertebrate genomes (including human). The larger the score, the more conserved

the site. Scores range from 0 to 1.

114 phastCons100way_vertebrate_rankscore: phastCons100way_vertebrate scores were ranked among

all phastCons100way_vertebrate scores in dbNSFP. The rankscore is the ratio of the rank

of the score over the total number of phastCons100way_vertebrate scores in dbNSFP.

115 phastCons20way_mammalian: phastCons conservation score based on the multiple alignments

of 20 mammalian genomes (including human). The larger the score, the more conserved

the site. Scores range from 0 to 1.

116 phastCons20way_mammalian_rankscore: phastCons20way_mammalian scores were ranked among

all phastCons20way_mammalian scores in dbNSFP. The rankscore is the ratio of the rank

of the score over the total number of phastCons20way_mammalian scores in dbNSFP.

117 SiPhy_29way_pi: The estimated stationary distribution of A, C, G and T at the site,

using SiPhy algorithm based on 29 mammals genomes.

118 SiPhy_29way_logOdds: SiPhy score based on 29 mammals genomes. The larger the score,

the more conserved the site. Scores range from 0 to 37.9718 in dbNSFP.

119 SiPhy_29way_logOdds_rankscore: SiPhy_29way_logOdds scores were ranked among all

SiPhy_29way_logOdds scores in dbNSFP. The rankscore is the ratio of the rank

of the score over the total number of SiPhy_29way_logOdds scores in dbNSFP.

120 1000Gp3_AC: Alternative allele counts in the whole 1000 genomes phase 3 (1000Gp3) data.

121 1000Gp3_AF: Alternative allele frequency in the whole 1000Gp3 data.

122 1000Gp3_AFR_AC: Alternative allele counts in the 1000Gp3 African descendent samples.

123 1000Gp3_AFR_AF: Alternative allele frequency in the 1000Gp3 African descendent samples.

124 1000Gp3_EUR_AC: Alternative allele counts in the 1000Gp3 European descendent samples.

125 1000Gp3_EUR_AF: Alternative allele frequency in the 1000Gp3 European descendent samples.

126 1000Gp3_AMR_AC: Alternative allele counts in the 1000Gp3 American descendent samples.

127 1000Gp3_AMR_AF: Alternative allele frequency in the 1000Gp3 American descendent samples.

128 1000Gp3_EAS_AC: Alternative allele counts in the 1000Gp3 East Asian descendent samples.

129 1000Gp3_EAS_AF: Alternative allele frequency in the 1000Gp3 East Asian descendent samples.

130 1000Gp3_SAS_AC: Alternative allele counts in the 1000Gp3 South Asian descendent samples.

131 1000Gp3_SAS_AF: Alternative allele frequency in the 1000Gp3 South Asian descendent samples.

132 TWINSUK_AC: Alternative allele count in called genotypes in UK10K TWINSUK cohort.

133 TWINSUK_AF: Alternative allele frequency in called genotypes in UK10K TWINSUK cohort.

134 ALSPAC_AC: Alternative allele count in called genotypes in UK10K ALSPAC cohort.

135 ALSPAC_AF: Alternative allele frequency in called genotypes in UK10K ALSPAC cohort.

136 ESP6500_AA_AC: Alternative allele count in the African American samples of the

NHLBI GO Exome Sequencing Project (ESP6500 data set).

137 ESP6500_AA_AF: Alternative allele frequency in the African American samples of the

NHLBI GO Exome Sequencing Project (ESP6500 data set).

138 ESP6500_EA_AC: Alternative allele count in the European American samples of the

NHLBI GO Exome Sequencing Project (ESP6500 data set).

139 ESP6500_EA_AF: Alternative allele frequency in the European American samples of the

NHLBI GO Exome Sequencing Project (ESP6500 data set).

140 ExAC_AC: Allele count in total ExAC samples (60,706 samples)

141 ExAC_AF: Allele frequency in total ExAC samples

142 ExAC_Adj_AC: Adjusted Alt allele counts (DP >= 10 & GQ >= 20) in total ExAC samples

143 ExAC_Adj_AF: Adjusted Alt allele frequency (DP >= 10 & GQ >= 20) in total ExAC samples

144 ExAC_AFR_AC: Adjusted Alt allele counts (DP >= 10 & GQ >= 20) in African & African American

ExAC samples

145 ExAC_AFR_AF: Adjusted Alt allele frequency (DP >= 10 & GQ >= 20) in African & African American

ExAC samples

146 ExAC_AMR_AC: Adjusted Alt allele counts (DP >= 10 & GQ >= 20) in American ExAC samples

147 ExAC_AMR_AF: Adjusted Alt allele frequency (DP >= 10 & GQ >= 20) in American ExAC samples

148 ExAC_EAS_AC: Adjusted Alt allele counts (DP >= 10 & GQ >= 20) in East Asian ExAC samples

149 ExAC_EAS_AF: Adjusted Alt allele frequency (DP >= 10 & GQ >= 20) in East Asian ExAC samples

150 ExAC_FIN_AC: Adjusted Alt allele counts (DP >= 10 & GQ >= 20) in Finnish ExAC samples

151 ExAC_FIN_AF: Adjusted Alt allele frequency (DP >= 10 & GQ >= 20) in Finnish ExAC samples

152 ExAC_NFE_AC: Adjusted Alt allele counts (DP >= 10 & GQ >= 20) in Non-Finnish European ExAC

samples

153 ExAC_NFE_AF: Adjusted Alt allele frequency (DP >= 10 & GQ >= 20) in Non-Finnish European ExAC

samples

154 ExAC_SAS_AC: Adjusted Alt allele counts (DP >= 10 & GQ >= 20) in South Asian ExAC samples

155 ExAC_SAS_AF: Adjusted Alt allele frequency (DP >= 10 & GQ >= 20) in South Asian ExAC samples

156 ExAC_nonTCGA_AC: Allele count in total ExAC_nonTCGA samples (53,105 samples)

157 ExAC_nonTCGA_AF: Allele frequency in total ExAC_nonTCGA samples

158 ExAC_nonTCGA_Adj_AC: Adjusted Alt allele counts (DP >= 10 & GQ >= 20) in total ExAC_nonTCGA samples

159 ExAC_nonTCGA_Adj_AF: Adjusted Alt allele frequency (DP >= 10 & GQ >= 20) in total ExAC_nonTCGA samples

160 ExAC_nonTCGA_AFR_AC: Adjusted Alt allele counts (DP >= 10 & GQ >= 20) in African & African American

ExAC_nonTCGA samples

161 ExAC_nonTCGA_AFR_AF: Adjusted Alt allele frequency (DP >= 10 & GQ >= 20) in African & African American

ExAC_nonTCGA samples

162 ExAC_nonTCGA_AMR_AC: Adjusted Alt allele counts (DP >= 10 & GQ >= 20) in American ExAC_nonTCGA samples

163 ExAC_nonTCGA_AMR_AF: Adjusted Alt allele frequency (DP >= 10 & GQ >= 20) in American ExAC_nonTCGA samples

164 ExAC_nonTCGA_EAS_AC: Adjusted Alt allele counts (DP >= 10 & GQ >= 20) in East Asian ExAC_nonTCGA samples

165 ExAC_nonTCGA_EAS_AF: Adjusted Alt allele frequency (DP >= 10 & GQ >= 20) in East Asian ExAC_nonTCGA samples

166 ExAC_nonTCGA_FIN_AC: Adjusted Alt allele counts (DP >= 10 & GQ >= 20) in Finnish ExAC_nonTCGA samples

167 ExAC_nonTCGA_FIN_AF: Adjusted Alt allele frequency (DP >= 10 & GQ >= 20) in Finnish ExAC_nonTCGA samples

168 ExAC_nonTCGA_NFE_AC: Adjusted Alt allele counts (DP >= 10 & GQ >= 20) in Non-Finnish European ExAC_nonTCGA

samples

169 ExAC_nonTCGA_NFE_AF: Adjusted Alt allele frequency (DP >= 10 & GQ >= 20) in Non-Finnish European ExAC_nonTCGA

samples

170 ExAC_nonTCGA_SAS_AC: Adjusted Alt allele counts (DP >= 10 & GQ >= 20) in South Asian ExAC_nonTCGA samples

171 ExAC_nonTCGA_SAS_AF: Adjusted Alt allele frequency (DP >= 10 & GQ >= 20) in South Asian ExAC_nonTCGA samples

172 ExAC_nonpsych_AC: Allele count in total ExAC_nonpsych samples (45,376 samples)

173 ExAC_nonpsych_AF: Allele frequency in total ExAC_nonpsych samples

174 ExAC_nonpsych_Adj_AC: Adjusted Alt allele counts (DP >= 10 & GQ >= 20) in total ExAC_nonpsych samples

175 ExAC_nonpsych_Adj_AF: Adjusted Alt allele frequency (DP >= 10 & GQ >= 20) in total ExAC_nonpsych samples

176 ExAC_nonpsych_AFR_AC: Adjusted Alt allele counts (DP >= 10 & GQ >= 20) in African & African American

ExAC_nonpsych samples

177 ExAC_nonpsych_AFR_AF: Adjusted Alt allele frequency (DP >= 10 & GQ >= 20) in African & African American

ExAC_nonpsych samples

178 ExAC_nonpsych_AMR_AC: Adjusted Alt allele counts (DP >= 10 & GQ >= 20) in American ExAC_nonpsych samples

179 ExAC_nonpsych_AMR_AF: Adjusted Alt allele frequency (DP >= 10 & GQ >= 20) in American ExAC_nonpsych samples

180 ExAC_nonpsych_EAS_AC: Adjusted Alt allele counts (DP >= 10 & GQ >= 20) in East Asian ExAC_nonpsych samples

181 ExAC_nonpsych_EAS_AF: Adjusted Alt allele frequency (DP >= 10 & GQ >= 20) in East Asian ExAC_nonpsych samples

182 ExAC_nonpsych_FIN_AC: Adjusted Alt allele counts (DP >= 10 & GQ >= 20) in Finnish ExAC_nonpsych samples

183 ExAC_nonpsych_FIN_AF: Adjusted Alt allele frequency (DP >= 10 & GQ >= 20) in Finnish ExAC_nonpsych samples

184 ExAC_nonpsych_NFE_AC: Adjusted Alt allele counts (DP >= 10 & GQ >= 20) in Non-Finnish European ExAC_nonpsych

samples

185 ExAC_nonpsych_NFE_AF: Adjusted Alt allele frequency (DP >= 10 & GQ >= 20) in Non-Finnish European ExAC_nonpsych

samples

186 ExAC_nonpsych_SAS_AC: Adjusted Alt allele counts (DP >= 10 & GQ >= 20) in South Asian ExAC_nonpsych samples

187 ExAC_nonpsych_SAS_AF: Adjusted Alt allele frequency (DP >= 10 & GQ >= 20) in South Asian ExAC_nonpsych samples

188 gnomAD_exomes_AC: Alternative allele count in the whole gnomAD exome samples (123,136 samples)

189 gnomAD_exomes_AN: Total allele count in the whole gnomAD exome samples (123,136 samples)

190 gnomAD_exomes_AF: Alternative allele frequency in the whole gnomAD exome samples (123,136 samples)

191 gnomAD_exomes_AFR_AC: Alternative allele count in the African/African American gnomAD exome samples (7,652 samples)

192 gnomAD_exomes_AFR_AN: Total allele count in the African/African American gnomAD exome samples (7,652 samples)

193 gnomAD_exomes_AFR_AF: Alternative allele frequency in the African/African American gnomAD exome samples (7,652 samples)

194 gnomAD_exomes_AMR_AC: Alternative allele count in the Latino gnomAD exome samples (16,791 samples)

195 gnomAD_exomes_AMR_AN: Total allele count in the Latino gnomAD exome samples (16,791 samples)

196 gnomAD_exomes_AMR_AF: Alternative allele frequency in the Latino gnomAD exome samples (16,791 samples)

197 gnomAD_exomes_ASJ_AC: Alternative allele count in the Ashkenazi Jewish gnomAD exome samples (4,925 samples)

198 gnomAD_exomes_ASJ_AN: Total allele count in the Ashkenazi Jewish gnomAD exome samples (4,925 samples)

199 gnomAD_exomes_ASJ_AF: Alternative allele frequency in the Ashkenazi Jewish gnomAD exome samples (4,925 samples)

200 gnomAD_exomes_EAS_AC: Alternative allele count in the East Asian gnomAD exome samples (8,624 samples)

201 gnomAD_exomes_EAS_AN: Total allele count in the East Asian gnomAD exome samples (8,624 samples)

202 gnomAD_exomes_EAS_AF: Alternative allele frequency in the East Asian gnomAD exome samples (8,624 samples)

203 gnomAD_exomes_FIN_AC: Alternative allele count in the Finnish gnomAD exome samples (11,150 samples)

204 gnomAD_exomes_FIN_AN: Total allele count in the Finnish gnomAD exome samples (11,150 samples)

205 gnomAD_exomes_FIN_AF: Alternative allele frequency in the Finnish gnomAD exome samples (11,150 samples)

206 gnomAD_exomes_NFE_AC: Alternative allele count in the Non-Finnish European gnomAD exome samples (55,860 samples)

207 gnomAD_exomes_NFE_AN: Total allele count in the Non-Finnish European gnomAD exome samples (55,860 samples)

208 gnomAD_exomes_NFE_AF: Alternative allele frequency in the Non-Finnish European gnomAD exome samples (55,860 samples)

209 gnomAD_exomes_SAS_AC: Alternative allele count in the South Asian gnomAD exome samples (15,391 samples)

210 gnomAD_exomes_SAS_AN: Total allele count in the South Asian gnomAD exome samples (15,391 samples)

211 gnomAD_exomes_SAS_AF: Alternative allele frequency in the South Asian gnomAD exome samples (15,391 samples)

212 gnomAD_exomes_OTH_AC: Alternative allele count in other gnomAD exome samples (2,743 samples)

213 gnomAD_exomes_OTH_AN: Total allele count in other gnomAD exome samples (2,743 samples)

214 gnomAD_exomes_OTH_AF: Alternative allele frequency in other gnomAD exome samples (2,743 samples)

215 gnomAD_genomes_AC: Alternative allele count in the whole gnomAD genome samples (15,496 samples)

216 gnomAD_genomes_AN: Total allele count in the whole gnomAD genome samples (15,496 samples)

217 gnomAD_genomes_AF: Alternative allele frequency in the whole gnomAD genome samples (15,496 samples)

218 gnomAD_genomes_AFR_AC: Alternative allele count in the African/African American gnomAD genome samples (4,368 samples)

219 gnomAD_genomes_AFR_AN: Total allele count in the African/African American gnomAD genome samples (4,368 samples)

220 gnomAD_genomes_AFR_AF: Alternative allele frequency in the African/African American gnomAD genome samples (4,368 samples)

221 gnomAD_genomes_AMR_AC: Alternative allele count in the Latino gnomAD genome samples (419 samples)

222 gnomAD_genomes_AMR_AN: Total allele count in the Latino gnomAD genome samples (419 samples)

223 gnomAD_genomes_AMR_AF: Alternative allele frequency in the Latino gnomAD genome samples (419 samples)

224 gnomAD_genomes_ASJ_AC: Alternative allele count in the Ashkenazi Jewish gnomAD genome samples (151 samples)

225 gnomAD_genomes_ASJ_AN: Total allele count in the Ashkenazi Jewish gnomAD genome samples (151 samples)

226 gnomAD_genomes_ASJ_AF: Alternative allele frequency in the Ashkenazi Jewish gnomAD genome samples (151 samples)

227 gnomAD_genomes_EAS_AC: Alternative allele count in the East Asian gnomAD genome samples (811 samples)

228 gnomAD_genomes_EAS_AN: Total allele count in the East Asian gnomAD genome samples (811 samples)

229 gnomAD_genomes_EAS_AF: Alternative allele frequency in the East Asian gnomAD genome samples (811 samples)

230 gnomAD_genomes_FIN_AC: Alternative allele count in the Finnish gnomAD genome samples (1,747 samples)

231 gnomAD_genomes_FIN_AN: Total allele count in the Finnish gnomAD genome samples (1,747 samples)

232 gnomAD_genomes_FIN_AF: Alternative allele frequency in the Finnish gnomAD genome samples (1,747 samples)

233 gnomAD_genomes_NFE_AC: Alternative allele count in the Non-Finnish European gnomAD genome samples (7,509 samples)

234 gnomAD_genomes_NFE_AN: Total allele count in the Non-Finnish European gnomAD genome samples (7,509 samples)

235 gnomAD_genomes_NFE_AF: Alternative allele frequency in the Non-Finnish European gnomAD genome samples (7,509 samples)

236 gnomAD_genomes_OTH_AC: Alternative allele count in other gnomAD genome samples (491 samples)

237 gnomAD_genomes_OTH_AN: Total allele count in other gnomAD genome samples (491 samples)

238 gnomAD_genomes_OTH_AF: Alternative allele frequency in other gnomAD genome samples (491 samples)

239 clinvar_rs: rs number from the clinvar data set

240 clinvar_clnsig: clinical significance as to the clinvar data set. 0 - unknown, 1 - untested,

2 - Benign, 3 - Likely benign, 4 - Likely pathogenic, 5 - Pathogenic, 6 - drug response,

7 - histocompatibility. A negative score means the the score is for the ref allele

241 clinvar_trait: the trait/disease the clinvar_clnsig referring to

242 clinvar_golden_stars: ClinVar Review Status summary.

0 - no assertion criteria provided, 1 - criteria provided, single submitter,

2 - criteria provided, multiple submitters, no conflicts,

3 - reviewed by expert panel, 4 - practice guideline

243 Interpro_domain: domain or conserved site on which the variant locates. Domain

annotations come from Interpro database. The number in the brackets following

a specific domain is the count of times Interpro assigns the variant position to

that domain, typically coming from different predicting databases. Multiple entries

separated by ";".

244 GTEx_V6p_gene: target gene of the (significant) eQTL SNP

245 GTEx_V6p_tissue: tissue type of the expression data with which the eQTL/gene pair is detected

Note 1: Missing data is designated as '.'.

Columns of dbNSFP_gene:

Gene_name: Gene symbol from HGNC

Ensembl_gene: Ensembl gene id (from HGNC)

chr: Chromosome number (from HGNC)

246 Gene_old_names: Old gene symbol (from HGNC)

247 Gene_other_names: Other gene names (from HGNC)

248 Uniprot_acc(HGNC/Uniprot): Uniprot acc number (from HGNC and Uniprot)

249 Uniprot_id(HGNC/Uniprot): Uniprot id (from HGNC and Uniprot)

250 Entrez_gene_id: Entrez gene id (from HGNC)

251 CCDS_id: CCDS id (from HGNC)

252 Refseq_id: Refseq gene id (from HGNC)

253 ucsc_id: UCSC gene id (from HGNC)

254 MIM_id: MIM gene id (from HGNC)

255 Gene_full_name: Gene full name (from HGNC)

256 Pathway(Uniprot): Pathway description from Uniprot

257 Pathway(BioCarta)_short: Short name of the Pathway(s) the gene belongs to (from BioCarta)

258 Pathway(BioCarta)_full: Full name(s) of the Pathway(s) the gene belongs to (from BioCarta)

259 Pathway(ConsensusPathDB): Pathway(s) the gene belongs to (from ConsensusPathDB)

260 Pathway(KEGG)_id: ID(s) of the Pathway(s) the gene belongs to (from KEGG)

261 Pathway(KEGG)_full: Full name(s) of the Pathway(s) the gene belongs to (from KEGG)

262 Function_description: Function description of the gene (from Uniprot)

263 Disease_description: Disease(s) the gene caused or associated with (from Uniprot)

264 MIM_phenotype_id: MIM id(s) of the phenotype the gene caused or associated with (from Uniprot)

265 MIM_disease: MIM disease name(s) with MIM id(s) in "[]" (from Uniprot)

266 Trait_association(GWAS): Trait(s) the gene associated with (from GWAS catalog)

267 GO_biological_process: GO terms for biological process

268 GO_cellular_component: GO terms for cellular component

269 GO_molecular_function: GO terms for molecular function

270 Tissue_specificity(Uniprot): Tissue specificity description from Uniprot

271 Expression(egenetics): Tissues/organs the gene expressed in (egenetics data from BioMart)

272 Expression(GNF/Atlas): Tissues/organs the gene expressed in (GNF/Atlas data from BioMart)

273 Interactions(IntAct): Other genes (separated by ;) this gene interacting with (from IntAct).

Full information (gene name followed by Pubmed id in "[]") can be found in the ".complete"

table

274 Interactions(BioGRID): Other genes (separated by ;) this gene interacting with (from BioGRID)

Full information (gene name followed by Pubmed id in "[]") can be found in the ".complete"

table

275 Interactions(ConsensusPathDB): Other genes (separated by ;) this gene interacting with

(from ConsensusPathDB). Full information (gene name followed by Pubmed id in "[]") can be

found in the ".complete" table

276 P(HI): Estimated probability of haploinsufficiency of the gene

(from doi:10.1371/journal.pgen.1001154)

277 P(rec): Estimated probability that gene is a recessive disease gene

(from DOI:10.1126/science.1215040)

278 Known_rec_info: Known recessive status of the gene (from DOI:10.1126/science.1215040)

"lof-tolerant = seen in homozygous state in at least one 1000G individual"

"recessive = known OMIM recessive disease"

(original annotations from DOI:10.1126/science.1215040)

279 RVIS_EVS: Residual Variation Intolerance Score, a measure of intolerance of mutational burden,

the higher the score the more tolerant to mutational burden the gene is. Based on EVS (ESP6500) data.

from doi:10.1371/journal.pgen.1003709

280 RVIS_percentile_EVS: The percentile rank of the gene based on RVIS, the higher the percentile

the more tolerant to mutational burden the gene is. Based on EVS (ESP6500) data.

281 LoF-FDR_ExAC: "A gene's corresponding FDR p-value for preferential LoF depletion among the ExAC population.

Lower FDR corresponds with genes that are increasingly depleted of LoF variants." cited from RVIS document.

282 RVIS_ExAC: "ExAC-based RVIS; setting 'common' MAF filter at 0.05% in at least one of the six individual

ethnic strata from ExAC." cited from RVIS document.

283 RVIS_percentile_ExAC: "Genome-Wide percentile for the new ExAC-based RVIS; setting 'common' MAF filter at 0.05%

in at least one of the six individual ethnic strata from ExAC." cited from RVIS document.

284 GHIS: A score predicting the gene haploinsufficiency. The higher the score the more likely the gene is

haploinsufficient. (from doi: 10.1093/nar/gkv474)

285 ExAC_pLI: "the probability of being loss-of-function intolerant (intolerant of both heterozygous and

homozygous lof variants)" based on ExAC r0.3 data

286 ExAC_pRec: "the probability of being intolerant of homozygous, but not heterozygous lof variants"

based on ExAC r0.3 data

287 ExAC_pNull: "the probability of being tolerant of both heterozygous and homozygous lof variants"

based on ExAC r0.3 data

288 ExAC_nonTCGA_pLI: "the probability of being loss-of-function intolerant (intolerant of both heterozygous and

homozygous lof variants)" based on ExAC r0.3 nonTCGA subset

289 ExAC_nonTCGA_pRec: "the probability of being intolerant of homozygous, but not heterozygous lof variants"

based on ExAC r0.3 nonTCGA subset

290 ExAC_nonTCGA_pNull: "the probability of being tolerant of both heterozygous and homozygous lof variants"

based on ExAC r0.3 nonTCGA subset

291 ExAC_nonpsych_pLI: "the probability of being loss-of-function intolerant (intolerant of both heterozygous and

homozygous lof variants)" based on ExAC r0.3 nonpsych subset

292 ExAC_nonpsych_pRec: "the probability of being intolerant of homozygous, but not heterozygous lof variants"

based on ExAC r0.3 nonpsych subset

293 ExAC_nonpsych_pNull: "the probability of being tolerant of both heterozygous and homozygous lof variants"

based on ExAC r0.3 nonpsych subset

294 ExAC_del.score: "Winsorised deletion intolerance z-score" based on ExAC r0.3.1 CNV data

295 ExAC_dup.score: "Winsorised duplication intolerance z-score" based on ExAC r0.3.1 CNV data

296 ExAC_cnv.score: "Winsorised cnv intolerance z-score" based on ExAC r0.3.1 CNV data

297 ExAC_cnv_flag: "Gene is in a known region of recurrent CNVs mediated by tandem segmental duplications and

intolerance scores are more likely to be biased or noisy." from ExAC r0.3.1 CNV release

298 GDI: gene damage index score, "a genome-wide, gene-level metric of the mutational damage that has

accumulated in the general population" from doi: 10.1073/pnas.1518646112. The higher the score

the less likely the gene is to be responsible for monogenic diseases.

299 GDI-Phred: Phred-scaled GDI scores

300 Gene damage prediction (all disease-causing genes): gene damage prediction (low/medium/high) by GDI

for all diseases

301 Gene damage prediction (all Mendelian disease-causing genes): gene damage prediction (low/medium/high)

by GDI for all Mendelian diseases

302 Gene damage prediction (Mendelian AD disease-causing genes): gene damage prediction (low/medium/high)

by GDI for Mendelian autosomal dominant diseases

303 Gene damage prediction (Mendelian AR disease-causing genes): gene damage prediction (low/medium/high)

by GDI for Mendelian autosomal recessive diseases

304 Gene damage prediction (all PID disease-causing genes): gene damage prediction (low/medium/high)

by GDI for all primary immunodeficiency diseases

305 Gene damage prediction (PID AD disease-causing genes): gene damage prediction (low/medium/high)

by GDI for primary immunodeficiency autosomal dominant diseases

306 Gene damage prediction (PID AR disease-causing genes): gene damage prediction (low/medium/high)

by GDI for primary immunodeficiency autosomal recessive diseases

307 Gene damage prediction (all cancer disease-causing genes): gene damage prediction (low/medium/high)

by GDI for all cancer disease

308 Gene damage prediction (cancer recessive disease-causing genes): gene damage prediction (low/medium/high)

by GDI for cancer recessive disease

309 Gene damage prediction (cancer dominant disease-causing genes): gene damage prediction (low/medium/high)

by GDI for cancer dominant disease

310 LoFtool_score: a percential score for gene intolerance to functional change. The lower the score the higher

gene intolerance to functional change. For details please contact Dr. Joao Fadista(joao.fadista@med.lu.se)

311 SORVA_LOF_MAF0.005_HetOrHom: the fraction of individuals in the 1000 Genomes Project data (N=2504)

who are either Heterozygote or Homozygote of LOF SNVs whose MAF<0.005. This fraction is from

a method for ranking genes based on mutational burden called SORVA (Significance Of Rare VAriants).

Please see doi: 10.1101/103218 for details.

312 SORVA_LOF_MAF0.005_HomOrCompoundHet: the fraction of individuals in the 1000 Genomes Project data (N=2504)

who are either Compound Heterozygote or Homozygote of LOF SNVs whose MAF<0.005. This fraction is from

a method for ranking genes based on mutational burden called SORVA (Significance Of Rare VAriants).

Please see doi: 10.1101/103218 for details.

313 SORVA_LOF_MAF0.001_HetOrHom: the fraction of individuals in the 1000 Genomes Project data (N=2504)

who are either Heterozygote or Homozygote of LOF SNVs whose MAF<0.001. This fraction is from

a method for ranking genes based on mutational burden called SORVA (Significance Of Rare VAriants).

Please see doi: 10.1101/103218 for details.

314 SORVA_LOF_MAF0.001_HomOrCompoundHet: the fraction of individuals in the 1000 Genomes Project data (N=2504)

who are either Compound Heterozygote or Homozygote of LOF SNVs whose MAF<0.001. This fraction is from

a method for ranking genes based on mutational burden called SORVA (Significance Of Rare VAriants).

Please see doi: 10.1101/103218 for details.

315 SORVA_LOForMissense_MAF0.005_HetOrHom: the fraction of individuals in the 1000 Genomes Project data (N=2504)

who are either Heterozygote or Homozygote of LOF or missense SNVs whose MAF<0.005. This fraction is from

a method for ranking genes based on mutational burden called SORVA (Significance Of Rare VAriants).

Please see doi: 10.1101/103218 for details.

316 SORVA_LOForMissense_MAF0.005_HomOrCompoundHet: the fraction of individuals in the 1000 Genomes Project data (N=2504)

who are either Compound Heterozygote or Homozygote of LOF or missense SNVs whose MAF<0.005. This fraction is from

a method for ranking genes based on mutational burden called SORVA (Significance Of Rare VAriants).

Please see doi: 10.1101/103218 for details.

317 SORVA_LOForMissense_MAF0.001_HetOrHom: the fraction of individuals in the 1000 Genomes Project data (N=2504)

who are either Heterozygote or Homozygote of LOF or missense SNVs whose MAF<0.001. This fraction is from

a method for ranking genes based on mutational burden called SORVA (Significance Of Rare VAriants).

Please see doi: 10.1101/103218 for details.

318 SORVA_LOForMissense_MAF0.001_HomOrCompoundHet: the fraction of individuals in the 1000 Genomes Project data (N=2504)

who are either Compound Heterozygote or Homozygote of LOF or missense SNVs whose MAF<0.001. This fraction is from

a method for ranking genes based on mutational burden called SORVA (Significance Of Rare VAriants).

Please see doi: 10.1101/103218 for details.

319 Essential_gene: Essential ("E") or Non-essential phenotype-changing ("N") based on

Mouse Genome Informatics database. from doi:10.1371/journal.pgen.1003484

320 MGI_mouse_gene: Homolog mouse gene name from MGI

321 MGI_mouse_phenotype: Phenotype description for the homolog mouse gene from MGI

322 ZFIN_zebrafish_gene: Homolog zebrafish gene name from ZFIN

323 ZFIN_zebrafish_structure: Affected structure of the homolog zebrafish gene from ZFIN

324 ZFIN_zebrafish_phenotype_quality: Phenotype description for the homolog zebrafish gene

from ZFIN

325 ZFIN_zebrafish_phenotype_tag: Phenotype tag for the homolog zebrafish gene from ZFIN

Columns of dbscSNV1.1:

chr: chromosome number

pos: physical position on the chromosome as to hg19 (1-based coordinate)

ref: reference nucleotide allele (as on the + strand)

alt: alternative nucleotide allele (as on the + strand)

hg38_chr: chromosome number as to hg38

hg38_pos: physical position on the chromosome as to hg38 (1-based coordinate)

RefSeq?: whether the SNV is a scSNV according to RefSeq

Ensembl?: whether the SNV is a scSNV according to Ensembl

RefSeq_region: functional region the SNV located according to RefSeq

RefSeq_gene: gene name according to RefSeq

RefSeq_functional_consequence: functional consequence of the SNV according to RefSeq

RefSeq_id_c.change_p.change: SNV in format of c.change and p.change according to RefSeq

Ensembl_region: functional region the SNV located according to Ensembl

Ensembl_gene: gene id according to Ensembl

Ensembl_functional_consequence: functional consequence of the SNV according to Ensembl

Ensembl_id_c.change_p.change: SNV in format of c.change and p.change according to Ensembl

ada_score: ensemble prediction score based on ada-boost. Ranges 0 to 1. The larger the

score the higher probability the scSNV will affect splicing. The suggested cutoff for

a binary prediction (affecting splicing vs. not affecting splicing) is 0.6.

rf_score: ensemble prediction score based on random forests. Ranges 0 to 1. The larger the

score the higher probability the scSNV will affect splicing. The suggested cutoff for

a binary prediction (affecting splicing vs. not affecting splicing) is 0.6.

Note 1: Missing data is designated as '.'.

Note 2: Multiple annotations are separated by ';'

Please cite:

Liu X, Jian X, and Boerwinkle E. 2011. dbNSFP: a lightweight database of human

non-synonymous SNPs and their functional predictions. Human Mutation. 32:894-899.

Liu X, Wu C, Li C and Boerwinkle E. 2016. dbNSFP v3.0: A One-Stop Database of Functional

Predictions and Annotations for Human Non-synonymous and Splice Site SNVs.

Human Mutation. 37(3):235-241.

Contact:

Xiaoming Liu, Ph.D.

Assistant Professor,

Human Genetics Center,

School of Public Health,

The University of Texas Health Science Center at Houston.

Email: xmliu.uth{at}gmail.com

Changelog:

February 23, 2011: dbNSFP and search_dbNSFP v0.9 released.

April 4, 2011: A bug related to the prediction scores of MutationTaster is fixed. dbNSFP v1.0

released. A change to the chromosome search order of the search_dbNSFP. A readme file added.

search_dbNSFP v1.0 released.

May 30, 2011: dbNSFP and search_dbNSFP v1.1 released. Version 1.1 added the following entries:

rs numbers from UniSNP (a cleaned version of dbSNP build 129), allele frequency recorded in dbSNP,

allele frequency reported by 1000 Genomes Project, alternative gene names, descriptive gene name,

database cross references (gene IDs of HGNC, MIM, Ensembl and HPRD). The unziped database is 18Gb.

May 31, 2011: dbNSFP_light and search_dbNSFP_light v1.0 released. dbNSFP_light v1.0 is a light

version of dbNSFP, which contains less annotation entries but some additional 9,285,316 NSs that

are not in CCDS version 20090327. Scores of PhyloP, SIFT, Polyphen2, LRT and MutationTaster are

included but missing data are not imputed. Prediction of LRT and MutationTaster are also included,

as well as the omega estimated by LRT. The unziped database is 6Gb.

October 24, 2011: dbNSFP_light v1.1 and search_dbNSFP_light v1.1 released. dbNSFP v1.2 and

search_dbNSFP v1.2 released. The new versions added GERP++ neutral rates and RS scores.

October 25, 2011: dbNSFP v1.3 released. It added Uniprot ID, accession number and amino acid

position based on Polyphen-2 annotation. Users now can search amino acid change directly referring

to a Uniprot ID or accession number.

November 3, 2011: dbNSFP_light v1.2 released. It added Uniprot ID, accession number and amino acid

position based on Polyphen-2 annotation. Users now can search amino acid change directly referring

to a Uniprot ID or accession number.

November 10, 2011: A bug fixed in the companion search program for dbNSFP v1.3, which causes invalid

search using AA mutations with Uniprot ID or accession number.

December 16, 2011: dbNSFP_light v1.3 released. It updated SIFT scores (August, 2011 version) and

Polyphen-2 scores (May, 2011 version). Uniprot ID, accession number and amino acid position based

on the Polyphen-2 annotations have been updated too.

April 11, 2012: dbNSFP2.0b1_variant released. This is beta test version of the variant sub-database

of dbNSFP v2.0, which is rebuilt based on Gencode release 9 / Ensembl version 64.

June 2, 2012: dbNSFP v2.0b2 released. It includes both the dbNSFP_variant and dbNSFP_gene sub-databases.

Slight changes have been made to the Ensembl gene and transcript ids of dbNSFP_variant in order to be

compatible to other database sources.

July 2, 2012: dbNSFP v2.0b3 released. An additional 2.2 million splicing site SNPs have been added to

dbNSFP_variant. In the table those SNPs have missing (".") in aaref, aaalt and "-1" in aapos. There's

no change to the format of search input file.

August 28, 2012: The companion java search program search_dbNSFP20b3 is updated. Added features include

supporting vcf file as input file and options for output contents (columns).

October 27, 2012: dbNSFP v2.0b4 is released. A new functional prediction score MutationAssessor is added

(I thank Mr. Yevgeniy Antipin for his recommendation). Allele frequencies from ESP 5400 data set are

replaced by ESP 6500 data set.

February 25, 2013: dbNSFP v2.0 is released. A new functional prediction score FATHMM is added.

March 22, 2013: A bug which caused a lot of missing FATHMM scores has been fixed.

May 31, 2013: The source code of the companion Java search program is now available under the RECEX SHARED

SOURCE LICENSE.

October 3, 2013: dbNSFP v2.1 is released. MutationTaster and FATHMM scores have been updated. Converted

scores of SIFT, LRT, MutationTaster, MutationAssessor and FATHMM have been added. Columns of SIFT and FATHMM

predictions have been added. The gene database has also been updated. Database IDs are updated. GO Slim terms,

pathway and protein interaction information from the ConsensusPathDB, and list of essential and non-essential

genes (based on phenotypes of mouse homologs) have been added.

January 23, 2014: dbNSFP v2.2 is released. SIFT and FATHMM now have multiple scores corresponding to different

Ensembl ENSP ids and amino acid positions (aapos_SIFT and aapos_FATHMM). Accordingly, our companion search

program now supports SNP searches based on Ensembl ENSP ids and amino acid positions. A bug is fixed for a

small proportion of MutationTaster scores.

January 26, 2014: dbNSFP v2.3 is released. Two ensemble scores (RadialSVM and LR) and their predictions have

been added.

February 12, 2014: A bug was fixed in dbNSFP v2.2 and v2.3, which caused missing delimiters in columns

aapos_SIFT, SIFT_score_converted and SIFT_pred. (I thank Mr. Yevgeniy Antipin for his reminder).

March 5, 2014: dbNSFP v2.4 is released. A whole genome functional prediction score called CADD was added,

along with five more conservation scores (phyloP46way_primate, phyloP100way_vertebrate, phastCons46way_primate,

phastCons46way_placental, phastCons100way_vertebarate). To facilitate comparison between scores, we added rank

scores for most functional prediction scores and conservation scores, and replacing the "converted" scores in

the previous versions.

June 1, 2014: dbNSFP v2.5 is released. A new functional score VEST 3.0 has been added. We thank Dr. Karchin for

kindly providing the score. A bug that causes the MutationTaster score error since v2.1 for variants with a

prediction of "Polymorphism_automatic" has been fixed. We thank John McGuigan and James Ireland for reporting

this bug. As MutationTaster can also predict splicing change and other functional effects, in case a variant has

multiple predictions based on their different model, we took the most damaging score and prediction for dbNSFP.

July 26, 2014: dbNSFP v2.6 is released. rs numbers from dbSNP 141 have been added to the variant database files.

Mouse and zebra fish homolog genes and phenotypes have been added to the gene database file (I thank Alex Li for

his suggestion and helps). Trait_association(GWAS) was also updated. An attached database called dbscSNV is

available for download. It includes all potential human SNVs within splicing consensus regions (−3 to +8 at the

5’ splice site and −12 to +2 at the 3’ splice site), i.e. scSNVs, related functional annotations and two ensemble

prediction scores for predicting their potential of altering splicing. A manuscript describing those scores have

been submitted. search_dbNSFP26 now supports searching dbNSFP along with dbscSNV using option "-s".

September 12, 2014: dbNSFP v2.7 is released. Chromosomes and positions of human reference hg38 have been added.

search_ dbNSFP27.class now supports query dbNSFP using the positions based on hg38 with the "-v hg38" option.

clinvar (freeze 20140902) annotations have been added. Allele frequencies from 2303 exomes of African Americans

and 3203 exomes of European Americans from the Atherosclerosis Risk in Communities Study (ARIC) cohort study

have been added. As the columns for gene interactions in dbNSFP_gene table contain very long strings, especially

for gene UBC, which may cause problems when viewing the results in Excel, now we only report the number of

interacting genes in those columns. Full information is retained in the dbNSFP_gene.complete table.

November 21, 2014: dbNSFP v2.8 is released. COSMIC (Catalogue Of Somatic Mutations In Cancer) annotation have

been added. Pathway information from BioCarta and KEGG (old version) has been added to the dbNSFP2.8_gene. A bug

causing inconsistency between MutationTaster scores and MutationTaster_pred, which affects v2.5 to v2.7, has

been fixed. I thank Adam Novak for reporting this bug.

February 3, 2015: dbNSFP v2.9 is released. SIFT score has been updated to ensembl66 version. PROVEAN score

(Protein Variation Effect Analyzer) v1.1 has been added. I thank Yongwook Choi from jcvi for providing the SIFT

and PROVEAN scores. CADD score has been updated to 1.3 version. Please note the following copyright statement

for CADD: "CADD scores (http://cadd.gs.washington.edu/) are Copyright 2013 University of Washington and

Hudson-Alpha Institute for Biotechnology (all rights reserved) but are freely available for all academic,

non-commercial applications. For commercial licensing information contact Jennifer McCullar (mccullaj@uw.edu)."

Allele frequency v0.3 of ~60,706 unrelated individuals from The Exome Aggregation Consortium (ExAC) has been added.

ExAC data are released under a Fort Lauderdale Agreement. Please refer to http://exac.broadinstitute.org/terms

for terms of use. I also want to thank Dr. CS (Jonathan) Liu from Softgenetics for providing hosting space.

April 6, 2015: dbNSFP v3.0b1 is released. The core set of nsSNVs and ssSNVs has been rebuilt based on Gencode 22/

Ensembl 79 with human reference sequence hg38. Putative genes have been included. Genes with incomplete 5' have

been excluded (I thank Chris Gillies for reporting the issues for genes with incomplete 5' end.) Genes on

mitochondrial DNA have been included. Allele frequencies from the UK10K cohorts and genotypes of two Neanderthals

have been added. Some resources have been updated, including the MutationTaster (I thank Dr. Dominik Seelow

for kindly providing the scores), allele frequencies from the 1000 Genomes Project populations, ancestral alleles,

dbSNP, ClinVar and InterPro. The presentation of the prediction scores has been improved by adding columns for

the corresponding transcript/protein ids. PhyloP and PhastCons conservation scores based on hg19 have been

replaced by the scores based on hg38. Some resources have been dropped due to various reasons, including SLR

test statistic, UniSNP ids, allele frequencies from the ARIC cohorts and allele counts in COSMIC. dbNSFP_gene

has also been completely rebuilt using the up-to-date resources. Residual Variation Intolerance Scores (RVIS)

have been added. GO Slim terms have been replaced by full GO terms. Two branches of dbNSFP are now provided:

dbNSFP3.0b1a suitable for academic use, which includes all the resources, and dbNSFP3.0b1c suitable for commercial

use, which does not include VEST3 and CADD.

April 12, 2015: dbNSFP v3.0b2 is released. This update fixed the issues due to inconsistent mitochondrial reference

sequences used by different resources. I thank Dr. Lishuang Shen at MEEI for helping solving the issues. For

mitochondrial SNV, the pos (i.e. hg38) refers to the rCRS (GenBank: NC_012920) and hg19_pos refers to a YRI

sequence (GenBank: AF347015). The ancestral allele of mitochondrial SNV now comes from the Reconstructed Sapiens

Reference Sequence (RSRS, doi:10.1016/j.ajhg.2012.03.002). The affected content include ancestral alleles,

Neanderthal/Denisova genotypes and MutationTaster columns of the chrM file. The rankscores of MutationTaster has

also been updated to reflect the update of its chrM scores. dbscSNV has been updated to v1.1 and added hg38

positions liftovered from its hg19 positions. Using search_dbNSFP30b2a or search_dbNSFP30b2c you can search

dbscSNV1.1 along with dbNSFP v3.0b2 with either hg19 coordinates or hg38 coordinates.

August 3, 2015: dbNSFP v3.0 is released. Three new functional prediction scores (DANN, fathmm-MKL and fitCons) and

two conservation scores (phyloP20way_mammalian and phastCons20way_mammalian) have been added to dbNSFP v3.0a.

All five scores except DANN are also included in bNSFP v3.0c. For commercial application of DANN, please contact

Daniel Quang (dxquang@uci.edu). CADD scores have been updated to v1.3. I thank Dr. Xueqiu Jian and Kirill Prusov for

suggestions on README files. dbNSFP v3.0 will be integrated into our new whole genome annotation pipeline WGSA version

0.6. Please join our Email group for news and updates from dbNSFP.

Columns updated: CADD_raw (dbNSFP v3.0a only), CADD_raw_rankscore (dbNSFP v3.0a only), CADD_phred (dbNSFP v3.0a only).

New columns: DANN_score (dbNSFP v3.0a only), DANN_rankscore (dbNSFP v3.0a only), fathmm-MKL_coding_score,

fathmm-MKL_coding_rankscore, fathmm-MKL_coding_pred, fathmm-MKL_coding_group, integrated_fitCons_score,

integrated_fitCons_rankscore, integrated_confidence_value, GM12878_fitCons_score, GM12878_fitCons_rankscore,

GM12878_confidence_value, H1-hESC_fitCons_score, H1-hESC_fitCons_rankscore, H1-hESC_confidence_value, HUVEC_fitCons_score,

HUVEC_fitCons_rankscore, HUVEC_confidence_value.

November 24, 2015: dbNSFP v3.1 is released. Significant eQTLs from GTEx V6 has been added. dbSNP rs has been updated to

build 144. Gene expression information (rpkm of RNAseq) of 53 tissues from GTEx V6 has been added to dbNSFP_gene. Three

gene intolerance scores (RVIS based on ExAC r0.3, GDI and LoFtool) has been added to dbNSFP_gene.

March 20, 2016: dbNSFP v3.2 is released. Eigen score, Eigen PC score (doi: 10.1038/ng.3477) and GenoCanyon score

(doi:10.1038/srep10576) have been added. Allele frequencies of two commonly used subsets of ExAC data (nonTCGA

and nonpsych) have been added. Mutation Assessor scores have been updated to release 3. PhyloP7way_vertebrate

and PhastCons7way_vertebrate conservation scores have been updated to PhyloP100way_vertebrate and PhastCons100way_vertebrate,

repectively. rankscores have been updated accordingly. Ancestral alleles have been updated based on Ensembl 84.

dbSNP has been updated to build 146. Clinvar has been updated to 20160302. InterPro has been updated to v56.

Gene name cross-links, IntAct, Uniprot, GWAS catalog, BioGRID, GO, ConsensusPathDB, mouse genes and zebra fish

genes information for the dbNSFP_gene table have been updated.

November 30, 2016: dbNSFP v3.3 and v2.9.2 are released. M-CAP score (DOI: 10.1038/ng.3703) has been added. We thank Dr. Gill Bejerano

for providing the score. Eigen and Eigen PC scores have been updated to v1.1. dbSNP has been updated to v147. clinvar has

been updated to 20161101.

March 12, 2017: dbNSFP v3.4 and v2.9.3 are released. REVEL score ( doi: 10.1016/j.ajhg.2016.08.016) and MutPred score

(doi: 10.1093/bioinformatics/btp528) have been added. SORVA gene ranking scores (doi: 10.1101/103218) have been added to

gene annotation.

August 6, 2017: dbNSFP v3.5 is released. Allele frequencies from the exomes and genomes of the Genome Aggregation Database (gnomAD)

have been added. Interpro, dbSNP, clinvar, ancestral alleles, Altai Neanderthal genotypes, Denisova genotypes and GTEx eQTLs have been updated.

dbNSFP_gene has been rebuilt with updated annotations. Other changes to dbNSFP_gene include: Interactions columns now show the gene list

instead of the total number; GTEx gene expression annotations have been removed; LoF FDR p-value from RVIS has been added;

Genome-wide haploinsufficiency score (GHIS) has been added; LoF and CNV intolerance/tolerance scores based on ExAC data have been added.
